# Single-atom catalyst for high-performance methanol oxidation

**DOI:** 10.1038/s41467-021-25562-y

**Published:** 2021-09-02

**Authors:** Zhiqi Zhang, Jiapeng Liu, Jian Wang, Qi Wang, Yuhao Wang, Kai Wang, Zheng Wang, Meng Gu, Zhenghua Tang, Jongwoo Lim, Tianshou Zhao, Francesco Ciucci

**Affiliations:** 1 Department of Mechanical and Aerospace Engineering, The Hong Kong University of Science and Technology, Hong Kong, China; 2 Department of Chemistry, College of Science, Seoul National University, Seoul, South Korea; 3 Department of Materials Science and Engineering, Guangdong Provincial Key Laboratory of Energy Materials for Electric Power, Southern University of Science and Technology, Shenzhen, China; 4 Guangzhou Key Laboratory for Surface Chemistry of Energy Materials and New Energy Research Institute, School of Environment and Energy, South China University of Technology, Guangzhou Higher Education Mega Centre, Guangzhou, China; 5 Department of Chemical and Biological Engineering, The Hong Kong University of Science and Technology, Hong Kong, China

**Keywords:** Electrocatalysis, Fuel cells, Materials for energy and catalysis, Nanoscience and technology

## Abstract

Single-atom catalysts have been widely investigated for several electrocatalytic reactions except electrochemical alcohol oxidation. Herein, we synthesize atomically dispersed platinum on ruthenium oxide (Pt_1_/RuO_2_) using a simple impregnation-adsorption method. We find that Pt_1_/RuO_2_ has good electrocatalytic activity towards methanol oxidation in an alkaline media with a mass activity that is 15.3-times higher than that of commercial Pt/C (6766 vs. 441 mA mg^‒1^_Pt_). In contrast, single atom Pt on carbon black is inert. Further, the mass activity of Pt_1_/RuO_2_ is superior to that of most Pt-based catalysts previously developed. Moreover, Pt_1_/RuO_2_ has a high tolerance towards CO poisoning, resulting in excellent catalytic stability. Ab initio simulations and experiments reveal that the presence of Pt‒O_3f_ (3-fold coordinatively bonded O)‒Ru_cus_ (coordinatively unsaturated Ru) bonds with the undercoordinated bridging O in Pt_1_/RuO_2_ favors the electrochemical dehydrogenation of methanol with lower energy barriers and onset potential than those encountered for Pt‒C and Pt‒Ru.

## Abstract (web summary)

It is still challenging to engineer single-atom catalysts for electrocatalytic methanol oxidation. Here, the authors design Pt single atom supported on RuO2 for highly active methanol oxidation in contrast to the inert Pt single atom supported on carbon.

## Introduction

Platinum is the most effective element for anodic methanol oxidation reaction (MOR) in direct methanol fuel cells^1,2^. The electrocatalytic activity of Pt is highly dependent on its geometrical structure and the surrounding environment^1^. To improve MOR activity and reduce Pt loading, conventional strategies have focused on^2^ (i) tailoring the structure and/or morphology of Pt (e.g., by making hollow/framed^3^ or core-shelled^4^ Pt); and (ii) hybridizing Pt with other elements (e.g., Co^3^, Ni^5^, Sn^6^, Bi^7^, etc.). However, the Pt in these catalysts is usually assembled as a nanoparticle of diameter greater than 1 nm, leading to unsatisfactory mass activity. Furthermore, in MOR, Pt nanoparticles are susceptible to poisoning by adsorbed intermediates (CO_ads_)^5^, resulting in activity loss. Hence, developing new types of Pt-based MOR electrocatalysts with high activity and anti-poisoning capability is of both practical and fundamental significance.

Single-atom catalysts (SACs) are now emerging as a new class of catalysts with extraordinary activity towards many electrocatalytic reactions, including oxygen and hydrogen evolution, oxygen, CO_2_, and N_2_ reduction, and hydrogen and formic acid oxidation^8–12^. Pt SACs have utmost utilization of Pt atoms and good capability for CO oxidation^13^. However, the electrochemical dehydrogenation of methanol to CO in MOR requires at least three contiguous Pt atoms^14^. Further, it has been reported that SACs consisting of Pt single atoms supported on carbon nanotubes are inactive towards MOR^15^. Yet, we should note that these studies focused only on the Pt active centers rather than the entire catalysts, thereby neglecting the environment surrounding Pt. In this regard, enhancing the activity of single atomic Pt towards MOR is a scientifically significant and challenging topic.

For SACs, the atomic coordination of single atoms also plays an important role in determining the catalytic activity^16^. It has been shown that the electronic structure and coordination of the central single atoms can be adjusted by tuning the bonds between the single atoms and the substrate^17–20^. Herein, we designed two types of Pt SACs. Thanks to a simple adsorption–impregnation preparation method^21^, single Pt atoms were immobilized on RuO_2_ and carbon black (VXC-72) to obtain Pt_1_/RuO_2_ and Pt_1_/VXC-72, respectively. The Pt_1_/RuO_2_ SACs showed superb mass activity (6766 mA mg^‒1^_Pt_) and stability towards the MOR, far superior to those of most Pt-based catalysts developed to date. The MOR mechanism including the dehydrogenation of methanol and CO electrooxidation is further studied by density functional theory (DFT), confirming the experimental observation that the prepared SACs are active for the alcohol oxidation reaction. This finding suggests an approach of SACs for direct alcohol fuel cells.

## Results

### Structure characterization of Pt_1_/RuO_2_

**Fig. 1:**
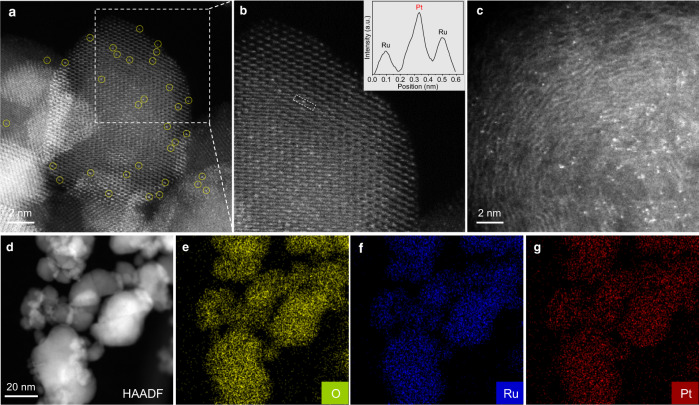
Morphological and elemental characterization of Pt_1_/RuO_2_ and Pt_1_/VXC-72. **a**, **b** Representative HAADF-STEM images of Pt_1_/RuO_2_. Inset in (**b**) is the intensity profile along with the dashed rectangle. **c** Representative HAADF-STEM image of Pt_1_/VXC-72. **d**–**g** EDS-mapping images of Pt_1_/RuO_2_.

RuO_2_ and VXC-72 supports were characterized by transmission electron microscopy (TEM), X-ray photoelectron spectroscopy (XPS), and X-ray diffraction (XRD) (Supplementary Fig. 1). Pt_1_/RuO_2_ and Pt_1_/VXC-72 catalysts with an identical Pt loading of 0.38 wt% were prepared by simple impregnation/adsorption followed by filtration and washing (for more details, see ‘Methods’ and Supplementary Fig. 2). Figure 1 shows the catalysts’ morphology and structure. As revealed by the bright spots marked as yellow circles in the high-angle annular dark-field (HAADF) scanning transmission electron microscopy (STEM) image, individual Pt atoms were randomly dispersed on RuO_2_ (Fig. 1a). The magnified HAADF-STEM image of Pt_1_/RuO_2_ suggests that the Pt atoms exactly substituted Ru (Fig. 1b). The presence of Pt single atoms was further verified by the intensity profile along with the dashed rectangle in the image (the inset in Fig. 1b), where the Pt atom brightness is more intense than that of Ru due to the higher Z number. The corresponding elemental mapping shows that Pt, Ru, and O were homogeneously distributed throughout the entire Pt_1_/RuO_2_ sample (Fig. 1d–g). For Pt_1_/VXC-72, Pt atoms were also atomically dispersed, as shown in the HAADF-STEM image (Fig. 1c). No Pt clusters/nanoparticles on VXC-72 were observed by low-magnification HAADF-STEM and TEM imaging (Supplementary Fig. 3). Due to the single-atom structure and low Pt loading, Pt peaks were, however, not detectable by XRD (Supplementary Fig. 4).

**Fig. 2:**
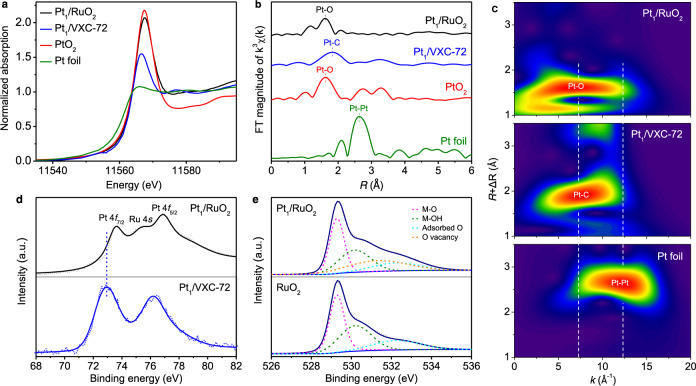
Structural characterization of Pt_1_/RuO_2_ and Pt_1_/VXC-72 by XAFS and XPS. **a** Normalized XANES spectra at the Pt *L*_3_-edge. **b** *k*^3^-weighted R-space Fourier transformed spectra from EXAFS. **c** Wavelet transforms from experimental data. **d** XPS spectra for Pt 4*f* and Ru 4*s*. **e** XPS spectra for O 1*s*. In panels (**a**–**c**) and (**e**), the corresponding data for Pt foil, PtO_2_, and RuO_2_ are presented for comparison.

To study the coordination and electronic configuration of Pt single atoms in the Pt_1_/RuO_2_ and Pt_1_/VXC-72, X-ray absorption fine structure (XAFS) spectroscopy and XPS were carried out (Fig. 2). The normalized X-ray absorption near-edge structure (XANES) spectra of Pt_1_/RuO_2_ shows stronger white-line intensity at 11,568 eV compared to the Pt foil, suggesting that Pt is positively charged in Pt_1_/RuO_2_ due to the electron transfer from Pt to the surrounding O atoms in the RuO_2_ support (Fig. 2a)^22,23^. The Pt_1_/VXC-72 catalyst is characterized by a similar Pt-to-VXC-72 electron transfer. However, the white-line peaks suggest that the Pt in Pt_1_/VXC-72 has a lower charge state than the one observed for Pt_1_/RuO_2_. The average Pt oxidation numbers in Pt_1_/RuO_2_ and Pt_1_/VXC-72 are estimated to be +2.90 and +1.22, respectively, by integrating the XANES spectra (Supplementary Fig. 5)^10,24^. The different charge of Pt in RuO_2_ and VXC-72 is likely a result of the different coordination. As revealed by the *k*^3^-weighted extended XAFS (EXAFS) at the Pt *L*_3_-edge, no peak at 2.64 Å from Pt‒Pt is observed for either Pt_1_/RuO_2_ or Pt_1_/VXC-72, indicating full atomic dispersion for both catalysts (Fig. 2b), consistent with the HAADF-STEM images. Specifically, the R-space spectrum of Pt in Pt_1_/RuO_2_ has a dominant peak at 1.62 Å from Pt‒O coordination, in agreement with the PtO_2_ case (Fig. 2b)^22,23^. In contrast, the dominant peak of Pt_1_/VXC-72 shifted to 1.83 Å (Fig. 2b). To determine the coordination of Pt in both catalysts, the main R-space peaks were fitted against corresponding models (see ‘Computational details’) by Fourier transform. For Pt_1_/RuO_2_, the coordination number of Pt‒O is estimated to be 4 with an average bond length of 1.99 Å (Supplementary Fig. 6 and Supplementary Table 1). For Pt_1_/VXC-72, the Pt‒C bond coordination number is 3, and the average bond length is 1.93 Å (Supplementary Fig. 6 and Supplementary Table 1). Also, the wavelet-transform plots of Pt_1_/RuO_2_ and Pt_1_/VXC-72 show a maximum at ~7.5 Å^−1^ corresponding to the Pt‒O/C bonds (Fig. 2c). No intensity maximum at ~12.5 Å^−1^ corresponding to the presence of Pt‒Pt bonds was observed for either catalyst, consistent with the EXAFS (Fig. 2c).

The RuO_2_ support contains Ru 4*s* with the binding energy at the range of ~70–85 eV (Supplementary Figs. 1b and 7), which is very close to the XPS signature of Pt 4*f*. Therefore, the Pt_1_/RuO_2_ XPS peaks in the ~68–82 eV range were deconvolved into Ru 4*s* and Pt 4*f*, i.e., Pt 4*f*_7/2_ and Pt 4*f*_5/2_ (Fig. 2d). The binding energies of the Pt 4*f* were measured to be 73.6 and 76.9 eV for Pt_1_/RuO_2_, and 72.9 and 76.2 eV for Pt_1_/VXC-72. The higher binding energy of Pt_1_/RuO_2_ compared to Pt_1_/VXC-72 implies that Pt in Pt_1_/RuO_2_ has a higher oxidation state, in agreement with the XANES results. Moreover, the O 1*s* spectrum of bare RuO_2_ was deconvolved into three characteristic peaks at 529.3, 530.2, and 532.3 eV (Fig. 2e), which can be attributed to M‒O, M‒OH, and adsorbed oxygen, respectively^25^. After the incorporation of Pt single atoms into RuO_2_, the O 1*s* of Pt_1_/RuO_2_ shows a new peak at  531.3 eV. This peak can be attributed to the appearance of oxygen vacancies on the surface resulting from Pt single atoms (Supplementary Fig. 8)^22,26^. These data support the existence of Pt‒O in Pt_1_/RuO_2_, in agreement with the XANES characterization. In addition, the O 1*s* of Pt_1_/VXC-72 is similar to that of VXC-72 (Supplementary Fig. 9), indicating that there is no Pt‒O interaction in Pt_1_/VXC-72. In other words, Pt‒C coordination is dominant in Pt_1_/VXC-72.

The preceding results indicate that Pt is atomically dispersed on both RuO_2_ and VXC-72 but surrounded by distinct coordination environments. Pt‒O coordination characterizes in Pt_1_/RuO_2_. In contrast, Pt‒C coordination is dominant in Pt_1_/VXC-72.

### MOR electrocatalytic activity

**Fig. 3:**
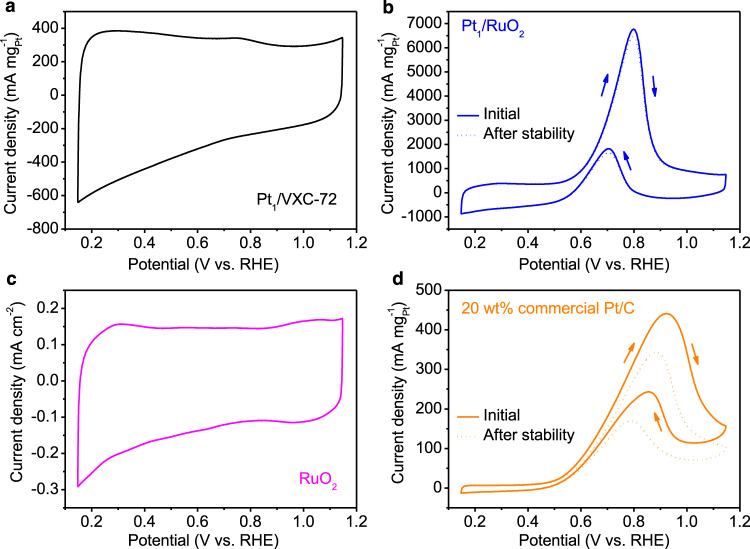
MOR performance of Pt_1_/RuO_2_ and control samples in 0.1 mol L^‒1^ KOH and 1 mol L^‒1^ methanol solution at a scan rate of 50 mV s^‒1^. **a** Pt_1_/VXC-72. **b** Pt_1_/RuO_2_. **c** RuO_2_. **d** 20 wt% commercial Pt/C.

Although Pt-based nanoparticles have been the most efficient catalysts for MOR, individual Pt atoms on carbon support are inactive towards that reaction^27^. By replacing the carbon support with RuO_2_, the coordination of Pt single atoms changes. Herein, we evaluated in the N_2_-saturated 0.1 mol L^‒1^ KOH and 1 mol L^‒1^ methanol solution the MOR performance of the Pt_1_/RuO_2_ SACs. Concomitantly, these materials were compared to Pt_1_/VXC-72 and commercial Pt/C, see Fig. 3. As revealed by cyclic voltammogram (CV) of Pt_1_/VXC-72 SACs, no electrocatalytic peak was detected, indicating that Pt_1_/VXC-72 is inactive towards MOR (Fig. 3a). We also prepared a Pt_1_/VXC-72 catalyst with a higher Pt loading of 1.48 wt%, i.e., Pt/VXC-72-1.48, which had a negligible MOR activity (Supplementary Fig. 10). These results support the literature findings that single atoms or even clusters of Pt on carbon are MOR inactive. Unexpectedly, the CV curves of Pt_1_/RuO_2_ SACs displayed oxidation current peaks in both the forward and backward CV scans (Fig. 3b), corresponding to methanol and intermediate products oxidation, respectively^7^. In contrast, the CV scan of the RuO_2_ was featureless (Fig. 3c). Further, oxygen vacancies on the RuO_2_ surface could not contribute to MOR (Supplementary Fig. 11). These results suggest that its MOR activity was due to having Pt single atoms on RuO_2_. The Pt mass activity of Pt_1_/RuO_2_ was 6766 mA mg^‒1^_Pt_ at 0.80 V vs. RHE (reversible hydrogen electrode), which is about 15.3 times higher than that of the commercial 20 wt% Pt/C (441 mA mg^‒1^_Pt_ at 0.92 V vs. RHE) (Fig. 3b, d) and significantly larger than the data for the reported catalysts to date (Supplementary Table 2). The peak current ratio between the forward (*I*_f_) and backward (*I*_b_) scan can be used to demonstrate the CO_ads_ tolerance^28^. The *I*_f_/*I*_b_ of Pt_1_/RuO_2_ (3.67) is more than twice that of Pt/C (1.81), suggesting an enhanced anti-poisoning ability for Pt_1_/RuO_2_. Long-term durability is another important criterion to assess the quality of a catalyst^5^. The stability of the Pt_1_/RuO_2_ SACs and 20 wt% commercial Pt/C was evaluated by chronoamperometry at ‒0.1 V (vs. Ag/AgCl). After 10 h, the Pt_1_/RuO_2_ mass activity remained at 6463 mA mg^‒1^_Pt_ with a slight degradation of 4.5% (Fig. 3b). In contrast, commercial Pt/C exhibited an ~22% decrease in mass activity (344 mA mg^‒1^_Pt_) (Fig. 3d). After the stability test, the Pt atoms in Pt_1_/RuO_2_ were isolated on the RuO_2_ support while the Pt nanoparticles in commercial 20 wt% Pt/C were aggregated (Supplementary Figs. 12 and 13).

**Fig. 4:**
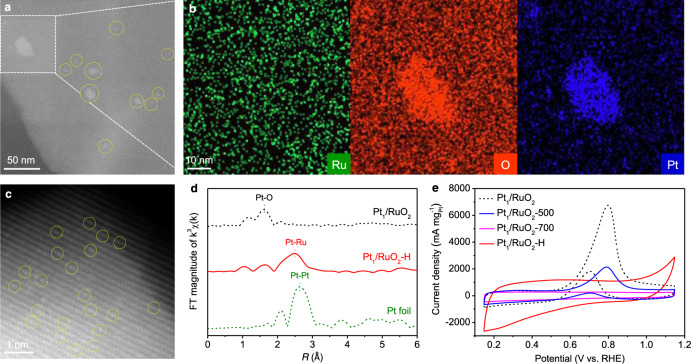
Characterizations and MOR performance of Pt_1_/RuO_2_-500/Pt_1_/RuO_2_-700 and Pt_1_/RuO_2_-H in 0.1 mol L^‒1^ KOH and 1 mol L^‒1^ methanol solution at a scan rate of 50 mV s^‒1^. **a** Typical HAADF-STEM images of Pt_1_/RuO_2_-700. **b** Corresponding EDS-mapping images of enlargement in (**a**). **c** Typical HAADF-STEM images of Pt_1_/RuO_2_-H. **d** *k*^3^-weighted R-space Fourier transformed spectra from EXAFS of Pt_1_/RuO_2_-H. **e** MOR performance. The curves in Figs. 2b and 3b are replotted here for convenient comparison.

To reveal the origin of MOR activity, several control samples were used, see Fig. 4. In the first set of samples denoted as Pt_1_/RuO_2_−500 and Pt_1_/RuO_2_−700, Pt_1_/RuO_2_ was annealed in air at 500 and 700 °C, respectively. After heat treatment, Pt single atoms on RuO_2_ were aggregated into nanoparticles with a 1–2 nm diameter for Pt_1_/RuO_2_−500, or even 30 nm diameter for Pt_1_/RuO_2_−700 (Supplementary Fig. 14 and Fig. 4a, b), respectively. When characterized by CV, Pt/RuO_2_−500 showed a mass activity of 2140 mA mg^‒1^_Pt_, a value lower than the 6766 mA mg^‒1^_Pt_ of Pt_1_/RuO_2_ SACs (Fig. 4e). For Pt/RuO_2_−700, the oxidation peak between 0.6 and 1.0 V disappeared, showing no MOR activity. The lower MOR performance of these catalysts can be attributed to the Pt aggregation. As is well known, catalytic activity is associated with the number of active sites^29^ and single atoms have many more of those compared to clusters or nanoparticles^30^. As expected, due to the presence of Pt clusters or nanoparticles, Pt/RuO_2_-0.75 and Pt/RuO_2_-1.48, two materials with higher Pt loading than Pt_1_/RuO_2_ SACs, showed decreased mass activity (Supplementary Fig. 15).

In parallel, an additional sample, denoted as Pt_1_/RuO_2_-H, was prepared by reducing the Pt_1_/RuO_2_ SACs in H_2_ at 80 °C. In that sample, the RuO_2_ support was reduced to metallic Ru with a little RuO_2_ left as confirmed by XRD (Supplementary Fig. 16). The different structure led to a changed coordination environment for Pt single atoms. The HAADF-STEM of Pt_1_/RuO_2_-H shows that the Pt atoms are isolated on the support (Fig. 4c). Compared to that of Pt_1_/RuO_2_, the EXAFS spectra of Pt_1_/RuO_2_-H show a prominent peak at 2.49 Å due to Pt‒Ru bond (coordination number *n* = 9), and the 1.62 Å peak of Pt‒O is negligible (Fig. 4d, Supplementary Fig. 17, and Supplementary Table 1)^31^. These results suggest that the Pt atoms have distinct coordination in Pt_1_/RuO_2_ and Pt_1_/RuO_2_-H, i.e., Pt‒O coordination for Pt_1_/RuO_2_ and Pt‒Ru for Pt_1_/RuO_2_-H. Accordingly, the Pt_1_/RuO_2_-H shows no MOR activity (Fig. 4e). Instead, Pt_1_/RuO_2_-H shows enhanced capacitive current and polarization current starting from ~0.9 V in the forward scan, resulting from higher relative Ru content in Pt_1_/RuO_2_-H than Pt_1_/RuO_2_. In consideration of the coordination of Pt‒O in Pt_1_/RuO_2_ and Pt‒C in Pt_1_/VXC-72, it can be concluded that the coordination of Pt single atoms governs MOR activity. On the basis of the preceding results, the origin of MOR activity in Pt_1_/RuO_2_ SACs can be attributed to: (i) the existence of large numbers of atomic Pt sites; and (ii) the environment surrounding Pt single atoms (thanks to RuO_2_, Pt single atoms turned into MOR-active catalysts).

**Fig. 5:**
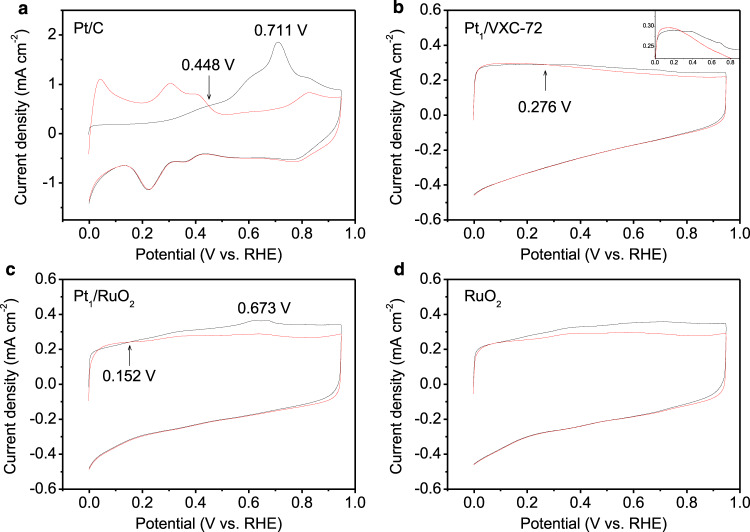
CO stripping tests in 0.1 mol L^‒1^ KOH at a scan rate of 50 mV s^‒1^. **a** 20 wt% commercial Pt/C. **b** Pt_1_/VXC-72. Inset in (**b**) is the local enlargement. **c** Pt_1_/RuO_2_. **d** RuO_2_. The black and red curves correspond to the first and second scans, respectively. The first scan was recorded in the presence of CO adsorbed on the electrode, while the second scan was recorded in absence of CO.

The CO resistibility of the catalysts was evaluated by CO stripping tests^6,9^, see Fig. 5. For commercial Pt/C, a main peak appeared in the first forward scan with onset and peak potentials at 0.448 and 0.711 V (Fig. 5a), respectively, where the latter value has been previously attributed to CO electrooxidation taking place on Pt nanoparticles^5^. Downsizing the Pt nanoparticles to the atomic scale in Pt_1_/VXC-72 reduces the onset potential of CO electrooxidation to ~0.276 V (Fig. 5b), a value much lower than that of commercial Pt/C (0.448 V). This reduction indicates that Pt single atoms trigger CO electrooxidation, consistent with the literature, where Pt single atoms have been shown to weaken CO adsorption, thereby facilitating the oxidation of CO at a lower potential^9^. Compared to Pt_1_/VXC-72 and commercial Pt/C, Pt_1_/RuO_2_ SACs triggered CO electrooxidation at a lower onset potential of 0.152 V with a minute peak around 0.673 V (Fig. 5c), suggesting an enhanced anti-poisoning capability for Pt_1_/RuO_2_. Such a change indicates that introducing RuO_2_ as a support favors CO electrooxidation. As is well known, oxophilic RuO_2_ boosts water dissociation, which, in turn, facilitates the formation of adsorbed OH and the oxidative removal of CO_ads_ on Pt sites, which, in turn, facilitates their regeneration^5,6^. As shown in Fig. 5d, RuO_2_ alone electrooxidized CO at an low potential of 0.076 V. Hence, both single atomic Pt and RuO_2_ favor the electrooxidative removal of CO, leading to the superior stability of Pt_1_/RuO_2_.

Ethanol oxidation reaction (EOR) tests in alkaline medium show an ~5.7-fold higher mass activity of Pt_1_/RuO_2_ compared to benchmark Pt/C (2824 vs. 498 mA mg^‒1^_Pt_) (Supplementary Fig. 18). These results suggest that Pt_1_/RuO_2_ also has remarkable potential for alcohol oxidation reactions.

### MOR mechanism

**Fig. 6:**
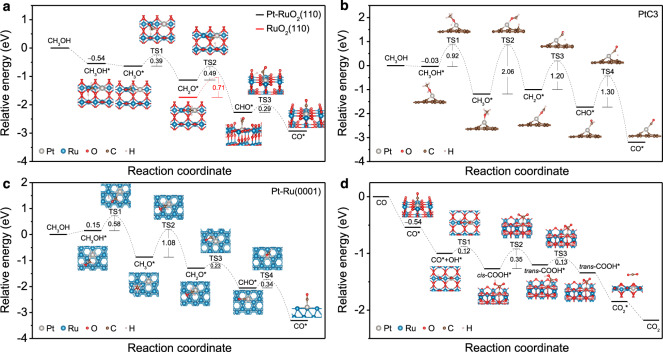
MOR mechanism. **a–c** Calculated reaction free energy and energy barriers for methanol oxidation to CO at: **a** Pt-RuO_2_(110), **b** PtC3, and **c** Pt-Ru(0001). **d** Calculated reaction free energy and energy barriers for CO oxidation on Pt-RuO_2_(110). In (**a**), the relative energies of CH_2_O* and corresponding TS2 computed for RuO_2_(110) are shown for reference.

To understand the MOR mechanism, three different models were constructed to simulate the catalytic reaction. The first system had a Pt atom located at the Ru_cus_ (1-fold coordinatively unsaturated Ru) site of the RuO_2_(110) surface (Pt-RuO_2_(110)) corresponding to Pt_1_/RuO_2_. The second model had a Pt atom bonded to three C atoms on graphene (PtC3) corresponding to Pt_1_/VXC-72. The final model had a Pt substituting a surface Ru in Ru(0001) (Pt-Ru(0001)) corresponding to Pt_1_/RuO_2_-H, see ‘Methods’ and Supplementary Figs. 19–21. The calculated MOR free energy diagrams of these three samples are shown in Fig. 6.

For the MOR on Pt-RuO_2_(110), the CH_3_OH molecule preferentially adsorbs on the site above Ru_cus_ with a free energy of ‒0.54 eV (Fig. 6a and Supplementary Fig. 22). Thanks to the highly active O_br_, the scission of O‒H bond of the adsorbed CH_3_OH molecule (CH_3_OH* → CH_3_O* + H*) proceeds spontaneously (Fig. 6a). For this elementary step, the cleaved H atom bonds to its neighboring O_br_ with a reaction free energy of ‒0.26 eV (Supplementary Fig. 22b and Supplementary Table 3). More importantly, the removal of H* (H* + OH^‒^ → H_2_O + e^‒^) to leave the sole adsorbate of CH_3_O* is slightly endothermic with a free energy requirement of 0.17 eV at pH = 13 (Supplementary Table 3). Considering that the O‒H bond scission is energetically favorable, we may propose that the MOR starts with the break of the O‒H bond in the methanol molecule and follows the CH_3_OH* → CH_3_O* → CH_2_O* → CHO* → CO* pathway^32,33^. As illustrated in Fig. 6a, the subsequent activations of the C‒H bond require energy barriers of 0.39 (CH_3_O* → TS1 → CH_2_O*), 0.49 (CH_2_O* → TS2 → CHO), and 0.29 eV (CHO* → TS3 → CO*), respectively. The dehydrogenation of CH_2_O* has been predicted to be the rate-limiting step for the above-mentioned pathway to CO. This result is consistent with DFT calculations from the Nørskov group showing that MOR on RuO_2_(110) is limited by the dehydrogenation of CH_2_O*^,^^34^. Nevertheless, the activation barrier on Pt-RuO_2_(110) was predicted to be only 0.49 eV, which is lower than the 0.71 eV we computed for pure RuO_2_(110) (Fig. 6a and Supplementary Fig. 23). This barrier reduction can be mainly attributed to the upshift in CH_2_O* energy due to Pt, which lowers the energy difference between the initial and the transition state of TS2. The final dehydrogenation of CHO (CHO* → TS3 → CO*) was predicted to have a barrier of 0.29 eV, implying that the oxidation of CHO is energetically favored compared to the other two reaction steps (CH_3_O* →TS1 → CH_2_O* and CH_2_O* → TS2 → CHO). The smaller barriers of all the above-mentioned reactions, in particular, the lower barrier of the dehydrogenation of CH_2_O* (CH_2_O* → TS2 → CHO), suggest that the introduction of Pt single atom into RuO_2_ improves the MOR kinetics. Both experimental evidence and DFT simulations support that the Pt‒O_3f_‒Ru configuration together with the O_br_ atoms is the active center, which is responsible for catalyzing the CH_3_OH into CO*.

The calculated free energy diagrams for MOR on PtC3 and Pt-Ru(0001) are shown in Fig. 6b, c for comparison. The adsorption free energy of CH_3_OH on the PtC3 and Pt-Ru(0001) are ‒0.03 and 0.15 eV, respectively. Both values are less negative than that computed for Pt-RuO_2_(110) (‒0.54 eV), implying a weaker interaction between both PtC3 and Pt-Ru(0001) and the methanol molecule in comparison to Pt-RuO_2_(110). Moreover, the energy barriers needed to cleave the O‒H and C‒H bonds are significantly higher than those of Pt-RuO_2_(110) (Fig. 6a–c). The much higher barriers compared to those on Pt-RuO_2_(110) indicate that the MOR is energetically costly for both PtC3 and Pt-Ru(0001) substrates. These computational results agree with experiments as both suggest that Pt_1_/VXC-72 and Pt_1_/RuO_2_-H are inert towards MOR. As for Pt-RuO_2_(101), the highest reaction activation barrier (0.79 eV) is much lower than that of both PtC3 (2.06 eV) and Pt-Ru(0001) (1.08 eV) (Supplementary Fig. 24 and Fig. 6b, c), suggesting the enhanced activity of Pt_1_/RuO_2_ compared to Pt_1_/VXC-72 and Pt_1_/RuO_2_-H towards MOR.

We also theoretically studied the stability of Pt-RuO_2_(110) with respect to CO poisoning, or equivalently, the oxidation CO into CO_2_. When OH* is present, CO oxidation proceeds through the CO* + OH* → TS1 → *cis*-COOH* → TS2 → *trans*-COOH* → TS3 → *trans*-COOH* → CO_2_* pathway^35^, as shown in Fig. 6d. The rate-limiting step of the overall reaction was predicted to be the *cis*-COOH* → *trans*-COOH* step (i.e., the rotation of the H atom in *cis*-COOH*) with activation barrier of 0.35 eV. This value is even smaller than the barrier computed for the dehydrogenation of formaldehyde on Pt-RuO_2_(110), implying that the presence of Pt facilitates the full oxidation of methanol into CO_2_ on RuO_2_(110). We further note that the *trans*-COOH* → CO_2_* step (i.e., the desorption of CO_2_*) has a positive Δ*G* of 0.33 eV, indicating that the release of CO_2_ is spontaneous. It is worth mentioning that the adsorption free energies of CO on the PtC3 and Pt-Ru(0001) are ‒0.81 and ‒0.92 eV (Fig. 6b, c), respectively. Both values are much more negative compared to that computed for Pt-RuO_2_(110) (‒0.54 eV), suggesting an increased interaction between CO and the PtC3/Pt-Ru(0001). According to the Brønsted-Evans-Polanyi relationship^36,37^, higher binding energy correlates to higher activation energies and lower thermodynamic driving force for the reaction. Therefore, we predict that the CO electrooxidation on PtC3 and Pt-Ru(0001) is much more energetically unfavorable with sluggish kinetics in agreement with the experiments above.

In addition to the activation energy barriers, onset potentials were also calculated (Supplementary Fig. 25). The potential-determining step (i.e., the elementary step with the highest onset potential) was found to be, for all three catalysts, the stripping off methoxyl proton (CH_3_O* → CH_2_O*). Accordingly, the onset potentials were predicted to be 0.26, 0.92, and 0.33 V for Pt-RuO_2_(110), PtC3, and Pt-Ru(0001), respectively. Both the lower onset potential and smaller activation barriers suggest that Pt_1_/RuO_2_ SACs are more favorable towards the methanol oxidation than Pt_1_/VXC-72 and Pt_1_/RuO_2_-H, in agreement with experimental results.

To unravel the mechanisms underpinning the high MOR activity, we calculated the projected density of states (PDOS) and charge analysis for both Pt and Ru_cus_ atoms in the Pt-RuO_2_(110). It is apparent that only the spin-up state of the *dz*^2^ orbital of Pt is filled while the *dz*^2^ orbital of Ru_cus_ is empty (Supplementary Fig. 26). Consistent with the PDOS results, the charge density difference also suggests that more electrons are depleted for the Ru_cus_ than the Pt atom (Supplementary Fig. 27). Specifically, Bader charge analysis suggests that Ru_cus_ and Pt on Pt-RuO_2_(110) lose 1.61 e and 1.22 e, respectively (Supplementary Table 4). Thanks to the empty anti-bonding *dz*^2^ orbital, a stronger interaction between the adsorbate (e.g., CH_3_OH) and Ru_cus_ than that of Pt is predicted, in agreement with the calculated adsorption free energies (Supplementary Fig. 22a–c). The orbital interaction between adsorbate, e.g., CH_3_OH, and the *dz*^2^ orbital is shown in Supplementary Fig. 28. In the case of Ru_cus_, for which the *dz*^2^ orbital is empty, the methanol molecule is well stabilized due to the favorable two-center two-electron interaction. Instead, the anti-bonding *dz*^2^ orbital of Pt is filled with one spin-up electron, resulting in the less-favorable two-center three-electron interaction, thereby destabilizing the adsorbate. Specifically, the filled anti-bonding Pt orbital leads to a weaker adsorption free energy of formaldehyde on Pt-RuO_2_(110) (‒0.26 eV) compared to that of RuO_2_(110) (‒0.88 eV) (Fig. 6a).The increased energy towards CH_2_O* thus lowers the activation barrier (TS2) for the subsequent dehydrogenation (Fig. 6a). We further evaluated the PDOS of the *d* orbitals with a Pt atom placed on different substrates. Compared to Pt-Ru(0001), the *d* orbitals of Pt on Pt-RuO_2_(110) are characterized by greater delocalization (Supplementary Fig. 29)^32^ as well as a larger density near the Fermi level. These features facilitate electron transfer and contribute to the enhanced MOR activity of Pt_1_/RuO_2_^35^. In contrast, the Pt PDOS of PtC3 is small at the Fermi level (Supplementary Fig. 29), suggesting that Pt_1_/VXC-72 is an ineffective MOR catalyst^9^. Besides, the additional *d* electrons in the Pt of PtC3 and Pt-Ru(0001) cause stronger repulsion to the coming ligand, e.g., CH_3_OH, thus weakening the adsorption of methanol and hindering MOR.

## Discussion

We successfully prepared Pt single atoms characterized by distinct coordination environment by leveraging a simple impregnation–adsorption method and tailoring the support. The Pt_1_/RuO_2_ SACs not only had a superb mass activity of 6766 mA mg^‒1^_Pt_ but also CO resistibility towards the MOR. Pt_1_/RuO_2_ was shown to be a significantly better catalyst compared to the commercial Pt/C benchmark. Theoretical simulations revealed that the Pt‒O_3f_‒Ru_cus_ with O_br_ in Pt_1_/RuO_2_ favored dehydrogenation of methanol with a low energy barrier and onset potential compared to that of Pt‒C and Pt‒Ru bonds. Moreover, CO at Pt_1_/RuO_2_ can be removed via electrooxidation at low potentials, leading to enhanced anti-poisoning capability. The discovery of Pt_1_/RuO_2_ SACs provides an approach to explore Pt SACs for MOR and related alcohol oxidation reaction.

## Methods

### Materials

H_2_PtCl_6_·6H_2_O, RuO_2_, and commercial Pt/C (20 wt% Pt) were purchased from Sigma-Aldrich. Carbon black (Vulcan XC-72) was bought from The Cabot Corporation. Methanol and ethanol were purchased from Scharlab.

### Synthesis of Pt_1_/RuO_2_ and control catalysts

The Pt_1_/RuO_2_ catalysts were prepared by an impregnation–adsorption method. Briefly, 50 mg of RuO_2_ were dispersed in 80 mL of distilled water. Next, 0.25 mL of a H_2_PtCl_6_·6H_2_O solution (2 mg mL^‒1^) was added dropwise; stirring at 70 °C for 5 h followed this. The product was obtained by filtration, repeated washing with water and ethanol, and drying at 70 °C overnight. Pt_1_/VXC-72 was prepared in a similar manner by using VXC-72. Pt_1_/RuO_2_-H catalyst was obtained by reducing Pt_1_/RuO_2_ in H_2_ at 80 °C. The Pt_1_/RuO_2_-500 and Pt_1_/RuO_2_-700 catalysts were obtained under the heat treatment of Pt_1_/RuO_2_ in the air at 500 and 700 °C, respectively.

### Catalyst characterization

The structure and morphology of the catalysts were characterized by XRD (PANalytical X’pert Pro, Cu Kα radiation), XPS (Axis Ultra DLD), and TEM (JEOL-2010F). The electron paramagnetic resonance (EPR) spectra were recorded at room temperature by using JEOL JES-FA200. The HAADF-STEM and EDS mapping were carried out using a double Cs-corrected FEI-Themis microscope operated at 300 kV. The STEM images were obtained with a convergent semi-angle of 25.1 mrad. The HAADF collection angle was 48–200 mrad. The XAFS at the Pt *L*_3_-edge was obtained on beamline 10C at the Pohang Light Source (PLS) in the Pohang Accelerator Laboratory (PAL), Republic of Korea. The XAFS data were processed and analyzed using ATHENA and ARTEMIS^38^. The Pt content in the catalyst was measured by ultraviolet visible spectroscopy (UV-vis, Lambda 20, Perkin Elmer). An appropriate amount of a H_2_PtCl_6_·6H_2_O solution (2 mg mL^‒1^) was added to deionized water and diluted to 100 mL. After the adsorption of Pt species by RuO_2_ in solution, the residual solution was measured by UV-vis, and by the comparison of the absorbance with the working curve, the Pt content in RuO_2_ was estimated.

### Electrochemical characterization

The electrochemical tests were conducted using a CHI 900D workstation (CH Instruments). A three-electrode setup was used and all tests were conducted at 25 °C. A graphite rod was used as a counter electrode; (KCl saturated) Ag/AgCl was selected as the reference electrode. To prepare the working electrode, 1 mg of catalyst and 1 mg of VXC-72 were ultrasonically dispersed in a mixture of 200 μL of water, 50 μL of ethanol, and 20 μL of Nafion (Dupont, 5 wt%). Next, 5 μL of the above ink was then placed onto a glassy-carbon electrode (4 mm in diameter) and dried at room temperature. In an N_2_-purged 0.1 mol L ^−1^ KOH and 1 mol L ^−1^ methanol/ethanol solution, the MOR/EOR electrocatalytic activity was evaluated by CV (scan rate was set at 50 mV s^−1^). CV scans were performed until a convergent response was recorded. For the CO stripping tests, CO was adsorbed on the catalyst by bubbling CO in 0.1 mol L ^−1^ KOH for 10 min at a potential of −0.95 V vs. Ag/AgCl. CO in the solution was eliminated by bubbling the electrolyte with N_2_ for 20 min. Stripping tests were carried out from −0.95 to 0 V at 50 mV s^−1^ for two consecutive scans. All measured potentials were converted to the RHE using *E*_RHE_ = *E*_Ag/AgCl_ + 0.198 + 0.059 × pH.

### Computational details

#### Models

The RuO_2_ substrate was modeled by the *p*(3 × 1) unit cell of RuO_2_(110) in a six-layered slab with 18 Ru atoms and 36 O atoms. The RuO_2_(110) was chosen because this surface has previously been reported to have the most thermodynamically favorable free energy during most synthesis procedures^34,39,40^. The RuO_2_(110) surface was constructed from the relaxed RuO_2_ bulk in the P4_2_/mnm space group, which has a lattice constant of *a* = *b* = 4.52 Å and *c* = 3.12 Å^41^. For a stoichiometric RuO_2_(110) surface, there are several different sites, i.e., the 1-fold coordinatively unsaturated Ru (Ru_cus_), the coordinatively saturated Ru (Ru_sat_), the undercoordinated bridging O (O_br_), and the 3-fold coordinatively bonded O (O_3f_) (Supplementary Fig. 19a). Among these sites, Ru_cus_ and O_br_ are widely believed to be catalytically active for chemical reactions^41,42^. To simulate the Pt_1_/RuO_2_, two different structures with one Pt atom located at either the Ru_cus_ or the Ru_sat_ of RuO_2_(110) were considered (Supplementary Fig. 19b). It was noticed that the Pt at the Ru_cus_ shows lower energy by 0.21 eV compared to that at the Ru_sat_, implying that the single Pt atom favors the Ru_cus_ (Supplementary Fig. 19b). A 5 × 5 graphene sheet and a five-layered slab of *p*(3 × 3) unit cell Ru(0001) were constructed to simulate the VXC-72 and the Ru substrate, respectively. One C atom in the graphene sheet and one top layer Ru atom in the Ru(0001) slab were replaced by the Pt to model the Pt_1_/VXC-72 and Pt_1_/RuO_2_-H, respectively (Supplementary Figs. 20 and 21). A vacuum space of 15 Å along the *c* direction was added for all substrates to avoid strong interactions between neighboring substrates.

#### DFT calculations

All the spin-polarized first-principle calculations were performed using the Vienna Ab initio Simulation Package (VASP)^43,44^ with a plane-wave basis set defined by a kinetic energy cutoff 400 eV. The projector augmented wave (PAW)^45^ pseudopotentials with valence-electron configurations of 2*s*^2^2*p*^2^, 2*s*^2^2*p*^4^, 1*s*^1^, 4*p*^6^4*d*^7^5*s*^1^, and 5*d*^9^6*s*^1^ were employed for C, O, H, Ru, and Pt, respectively. The electron exchange-correlation was described using the Perdew-Burk-Ernzerhof (PBE)^46^ functional under the generalized gradient approximation (GGA) scheme. For all the calculations, a dipole correction along the *c* direction was also applied. The bottom three layers of the RuO_2_(110) and the Ru(0001) slabs were fixed during the relaxation, whereas the top three layers were fully relaxed until the energy and force converged within 10^−5^ eV and 0.02 eV Å^−1^, respectively. The Brillouin zones were sampled using Gamma centered *k-*points with spacing smaller than 2π × 0.04 Å^−1^.

The binding energy of one Pt atom with the substrate was evaluated as *E*_bind_ = *E*(Pt-sub) ‒ *E*(Pt) ‒ *E*(sub), where *E*(Pt-sub) and *E*(sub) are the energies of the system with one Pt atom and the pure substrate, respectively, and *E*(Pt) is the total energy per atom for Pt bulk metal. The transition states (TSs) were located by the combination of both the nudged elastic band (NEB) method and the dimer method^47–49^. All the TSs were confirmed by noticing only one imaginary vibrational frequency. The adsorption energy of the intermediate was calculated by Δ*E*_ads_ = *E*(adsorbate-sub) ‒ *E*(sub) ‒ *E*(adsorbate), where *E*(adsorbate-sub) is the energy of the system with the adsorbate on the substrate, *E*(sub) is the energy of the clean substrate, and *E*(adsorbate) is the energy of the isolated adsorbate molecule in the vacuum. The adsorption free energy was further calculated as Δ*G*_ads_ = Δ*E*_ads_ + ΔZPE ‒ *T*Δ*S*, where ΔZPE is the change of zero-point energies, and Δ*S* is the entropy difference, and *T* is the temperature (298.15 K). The activation energy was calculated as *E*_a_ = *E*(TS) ‒ *E*(IS), where *E*(TS) and *E*(IS) are the energies for the TS and the initial state (IS).

The change in Gibbs free energy (Δ*G*) for all the reaction steps were calculated based on the method developed by Nørskov et al.^50^ At experimental condition (*U* = 0, pH, *p* = 1 bar, *T* = 298 K), the free energy Δ*G* is calculated as Δ*G* = Δ*E* + ΔZPE − *T*Δ*S* + Δ*G*_pH_, where Δ*E* is the energy difference by DFT calculations, and Δ*G*_pH_ corresponds to the correction due to the pH that is different from 0, i.e., Δ*G*_pH_ = − *k*_B_*T* ln[H^+^] = 0.0592 pH. The free energy of OH^−^ was calculated by *G*(OH^−^) = *G*(H_2_O(l)) – *G*(H^+^) = *G*(H_2_O(l)) – *G*(H_2_(g))/2^51^, where *G*(H_2_O(l)) is the Gibbs free energy of one water molecule under the saturated vapor pressure (0.035 bar), and *G*(H^+^) is taken as half of *G*(H_2_(g)), i.e., the free energy of H_2_(g) at standard condition (*p* = 1 bar, *T* = 298 K).

The computational standard hydrogen electrode was used to calculate the onset potentials^33,50^. Under this framework, the influence of the applied potential *U* was computed by adding ∆*G*_*U*_ = ‒e*U* to the free energy of a reaction involving the formation of a proton–electron pair. The electrochemical potential pertaining to each elementary step was calculated using *U*_*i*_ = ∆*G*_*i*_*/*e, where ∆*G*_*i*_ is the reaction free energy calculated according to Supplementary Table 5. The overall onset potential was estimated as *U*_onset_ = max(∆*G*_*i*_*/*e*)*^33^.

## Supplementary information

Supplementary information.

## Data Availability

All the raw data used in this work are available on reasonable request from the corresponding author.
