# Supplementary material for: Single-atom catalyst for high-performance methanol oxidation
Source: Nat Commun. 2021 Sep 2;12:5235. doi: 10.1038/s41467-021-25562-y (PMC8413426; doi:10.1038/s41467-021-25562-y)
Supplement: Supplementary file 1 — Supplementary information. [file 41467_2021_25562_MOESM1_ESM.pdf]

Supplementary Information

Single-atom catalyst for high-performance methanol oxidation

Zhiqi Zhang et al.

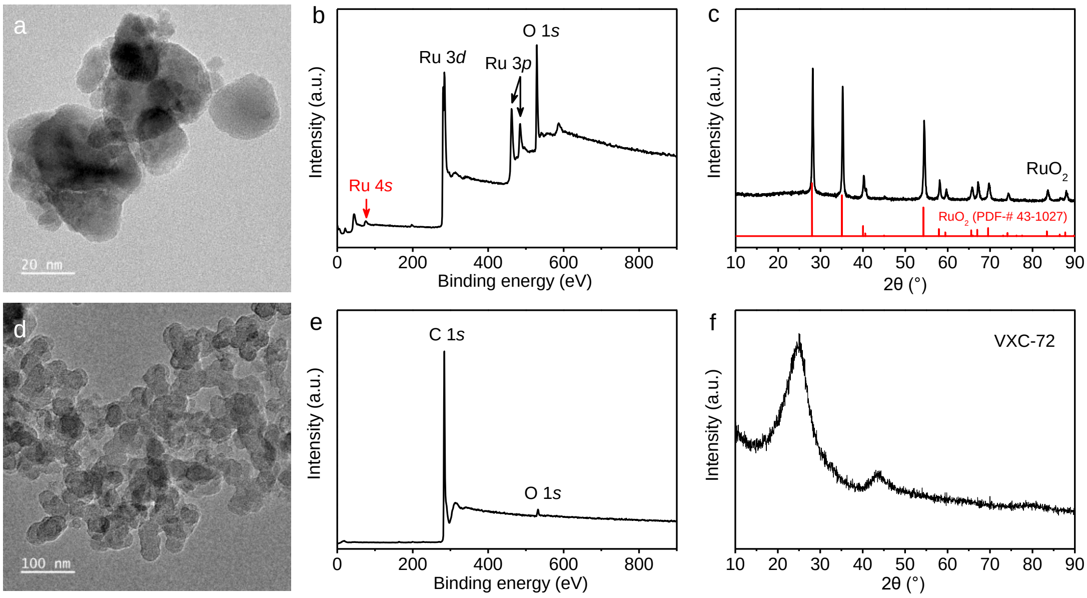

Supplementary Figure 1. Characterizations of RuO_2 and VXC-72. (a) TEM image, (b) XPS survey scan, and (c) XRD pattern of RuO_2. (d) TEM image, (e) XPS survey scan, and (f) XRD pattern of VXC-72.

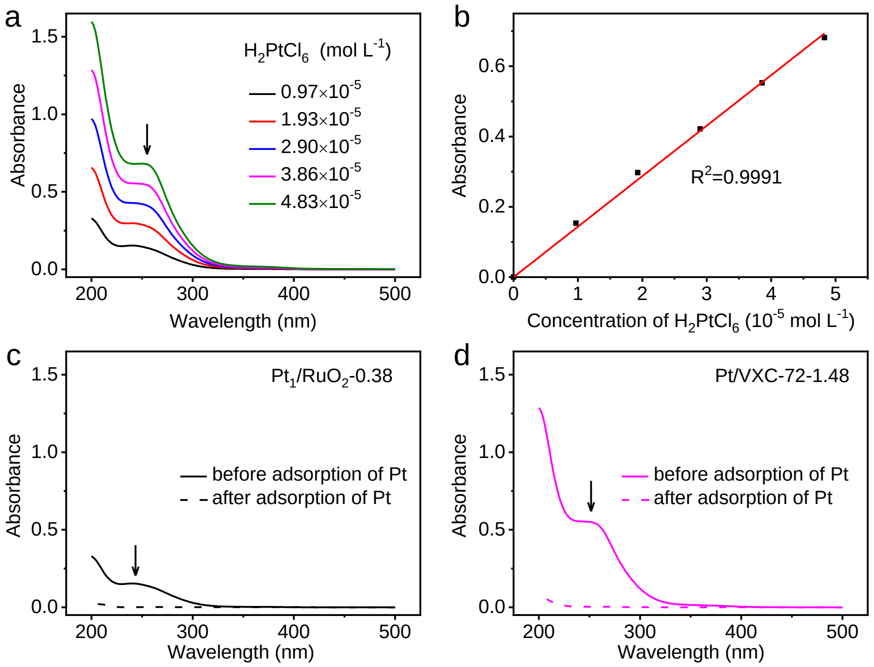

Supplementary Figure 2. UV-vis absorption measurements. (a) UV-vis spectra of the H_2PtCl_6 (aqueous) solutions used for (b) H_2PtCl_6 concentration versus absorbance. (c, d) UV-vis spectra of the solution before and after adsorption of Pt species for (c) Pt_1/RuO_2-0.38 and (d) Pt/VXC-72-1.48.

After adsorption on the RuO_2 or VXC-72 supports in solution, the Pt in the aqueous solution was not detectable (Supplementary Fig. 2c, d), suggesting that Pt was completely adsorbed on the supports. Hence, the Pt loading was calculated accordingly (See Methods).

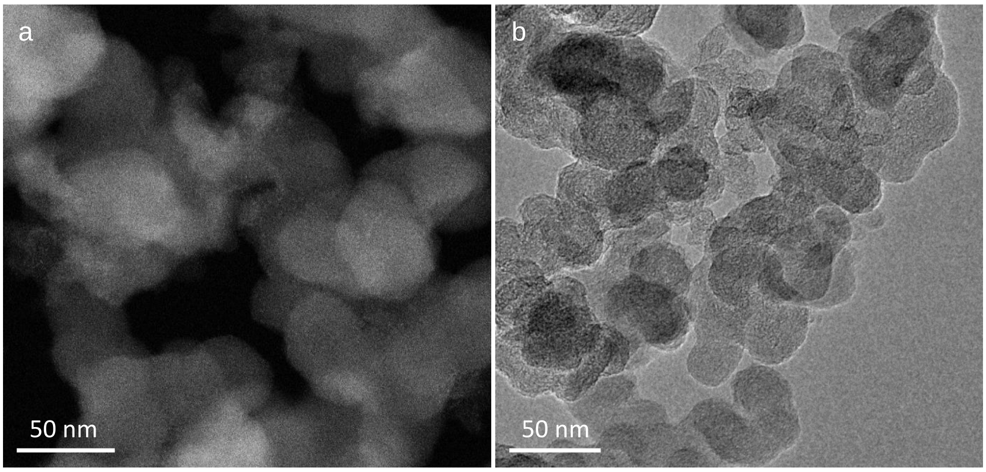

Supplementary Figure 3. Characterizations of Pt_1/VXC-72. (a) Low-magnification HAADF-STEM image. (b) TEM image.

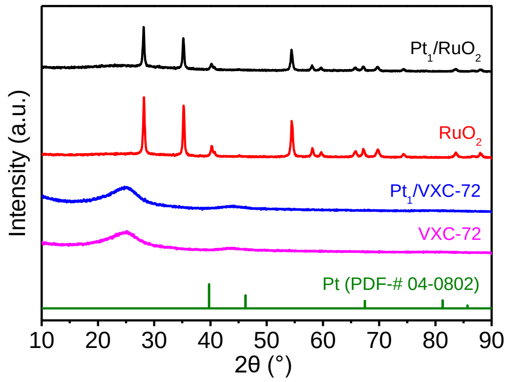

Supplementary Figure 4. XRD patterns of Pt_1/RuO_2, Pt_1/VXC-72, RuO_2, and VXC-72.

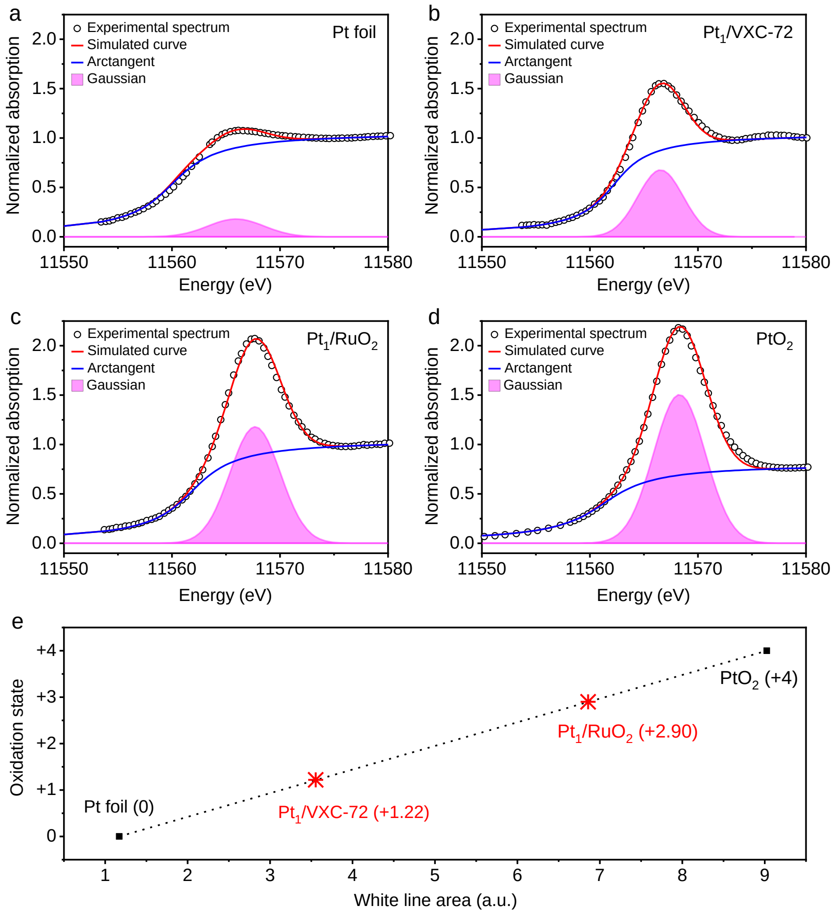

Supplementary Figure 5. Quantitative determination of the average oxidation state of Pt. (a, b, c, d) Fitting analysis of Pt L_3-edge XANES spectra peak of Pt foil, Pt_1/VXC-72, Pt_1/RuO_2, and PtO_2 by an arctangent function and a Gauss function, respectively. (e) Fitted average oxidation state of Pt in Pt_1/VXC-72 and Pt_1/RuO_2 from XANES spectra. Pt foil (0) and PtO_2 (+4) were employed as standard references.

By arctangent function as baseline and Gaussian function as fitted curves, the Pt L_3-edge XANES spectra peak of Pt foil and PtO_2 references as well as Pt_1/VXC-72 and Pt_1/RuO_2 are fitted (Supplementary Fig. 5a-d)[1,2]. As is well known, the valence state of Pt in Pt foil and PtO_2 references is 0 and +4, respectively. Due to the linear relationship of the white line area and the Pt oxidation state[1,2], the oxidation states of Pt in Pt_1/VXC-72 and Pt_1/RuO_2 can be determined, that is, +1.22 and +2.90, respectively (Supplementary Fig. 5e).

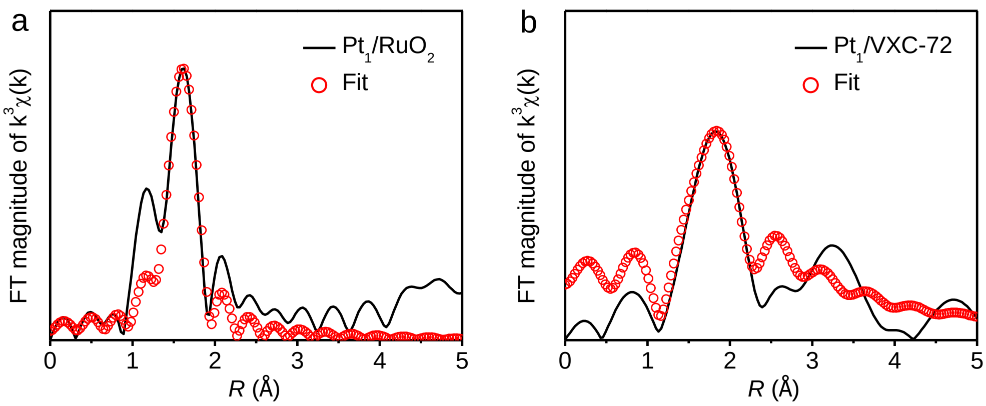

Supplementary Figure 6. EXAFS R-space experimental curve and fitting curve against models of Pt-RuO_2(110) and PtC3 (see Supplementary Fig. 19b, 20) for (a) Pt_1/RuO_2 and (b) Pt_1/VXC-72, respectively. The fit parameters are shown in Supplementary Table 1.

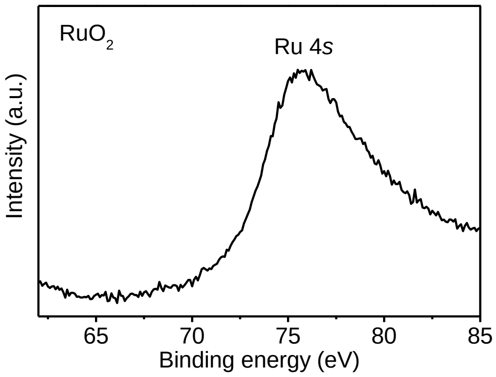

Supplementary Figure 7. Ru 4*s* XPS spectra of RuO_2.

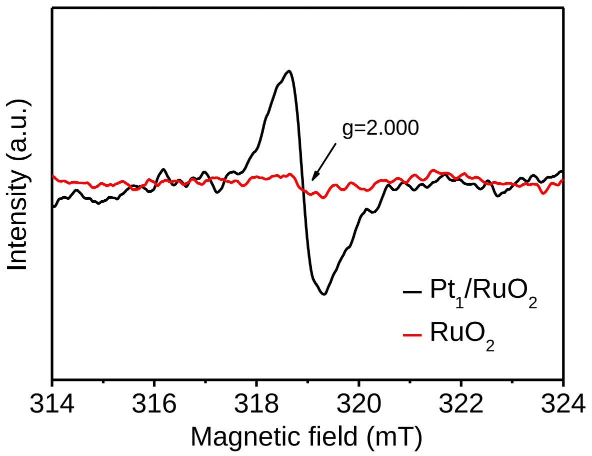

Supplementary Figure 8. EPR spectra of Pt_1/RuO_2 and RuO_2.

The signal intensity of oxygen vacancies of Pt_1/RuO_2 is higher than that of RuO_2, suggesting the existence of oxygen vacancies as a consequence of the presence of Pt single atoms[3].

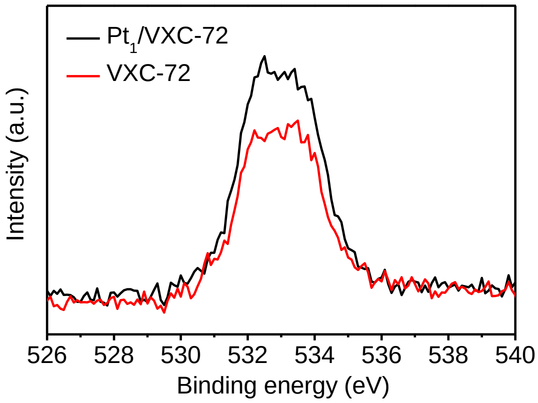

Supplementary Figure 9. O 1*s* XPS spectra of Pt_1/VXC-72 and VXC-72.

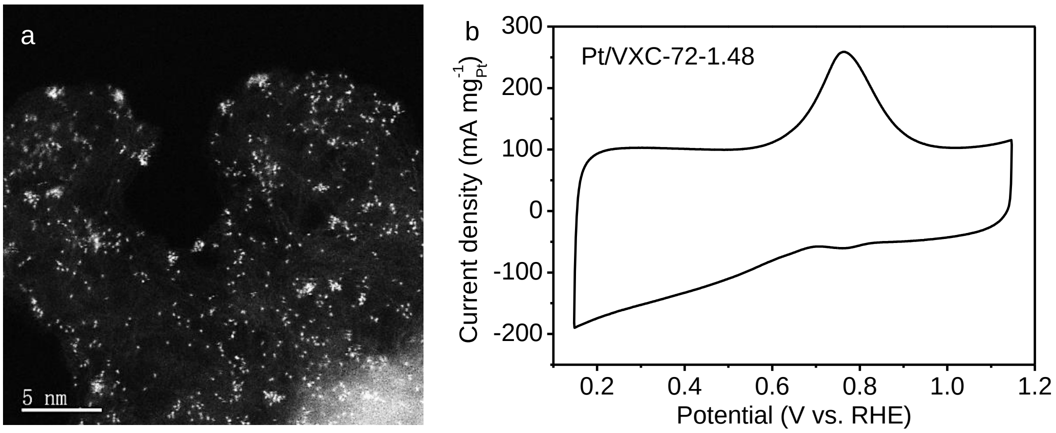

Supplementary Figure 10. HAADF-STEM image (a) of Pt/VXC-72-1.48 and corresponding MOR performance (b).

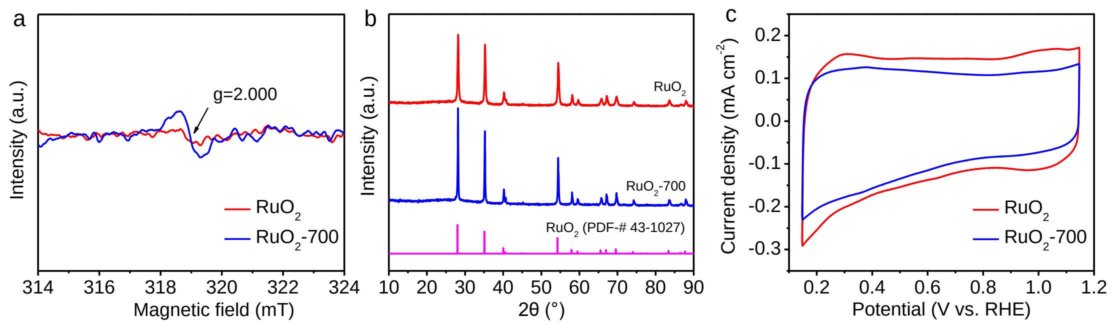

Supplementary Figure 11. (a) EPR spectra, (b) XRD patterns, and (c) MOR performance of RuO_2 and RuO_2-700 in 0.1 mol L^{-1} KOH and 1 mol L^{-1} methanol solution at a scan rate of 50 mV s^{-1}.

To reveal the role of oxygen vacancy during the MOR, we prepared a control sample that RuO_2 annealed in air at 700 °C (denoted as RuO_2-700). The RuO_2-700 remained RuO_2 phase but showed an enhanced signal intensity of oxygen vacancies compared to pristine RuO_2 (Supplementary Fig. 11a, b). However, RuO_2-700 still had no MOR performance (Supplementary Fig. 11c), suggesting the negligible contribution of oxygen vacancies in RuO_2 to MOR.

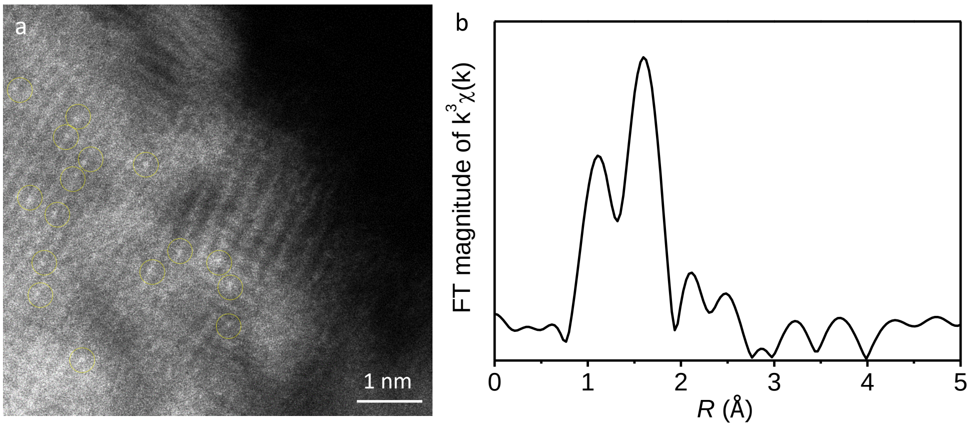

Supplementary Figure 12. Characterizations of Pt_1/RuO_2 after stability test. (a) HAADF-STEM image. (b) k^3-weighted R-space Fourier transformed spectra from EXAFS.

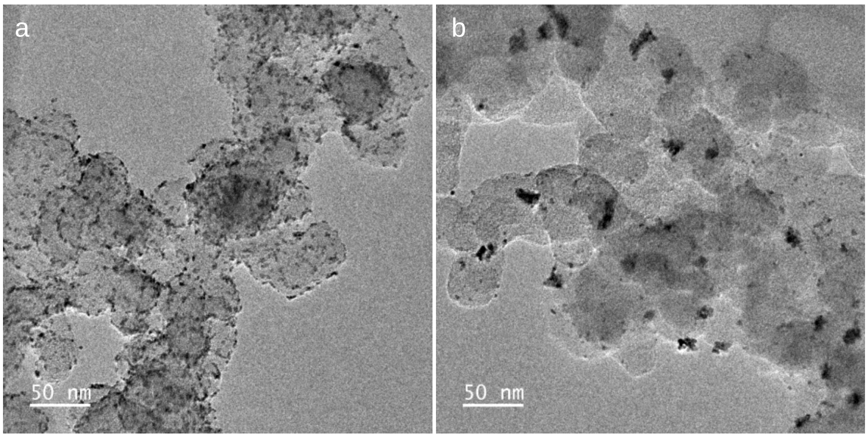

Supplementary Figure 13. TEM images of 20 wt% commercial Pt/C. (a) Before stability test. (b) After stability test.

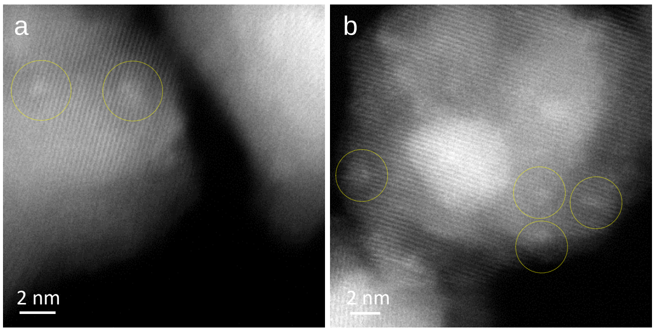

Supplementary Figure 14. HAADF-STEM images of Pt_1/RuO_2-500.

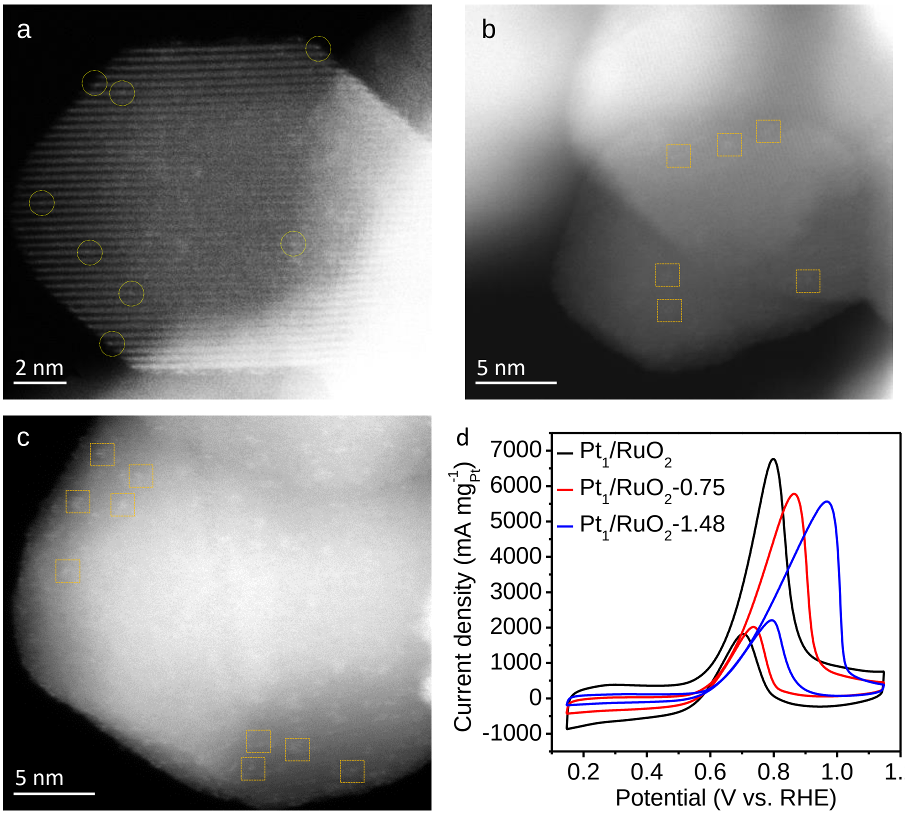

Supplementary Figure 15. HAADF-STEM images of (a, b) Pt/RuO_2-0.75, (c) Pt/RuO_2-1.48, and (d) MOR performance in 0.1 mol L^{-1} KOH and 1 mol L^{-1} methanol solution at a scan rate of 50 mV s^{-1}.

For the Pt/RuO_2-0.75 with Pt loading of 0.75 wt%, Pt mainly existed in single atoms and clusters (Supplementary Fig. 15a, b). For the higher Pt loading of 1.48 wt% as shown in Pt/RuO_2-1.48, Pt mainly existed in clusters and nanoparticles (Supplementary Fig. 15 c). With the increase of Pt loading on RuO_2, Pt atoms tended to aggregation, which can lead to the decrease in the numbers of activity sites towards electrocatalysis[4]. Correspondingly, the Pt/RuO_2-0.75 and Pt/RuO_2-1.48 showed mass activities of 5785 and 5575 mA mg_{Pt}^{-1} (Supplementary Fig. 15d), respectively, which were lower than that of Pt_1/RuO_2 SACs (6766 mA mg_{Pt}^{-1}) with 0.38 wt% Pt loading.

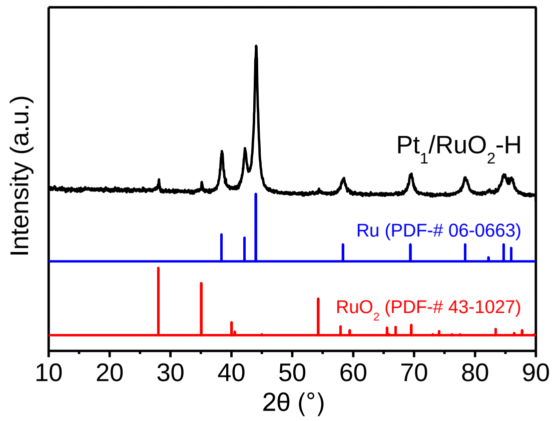

Supplementary Figure 16. XRD patterns of Pt_1/RuO_2-H.

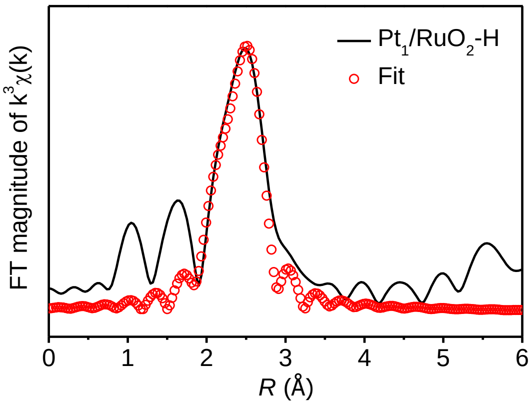

Supplementary Figure 17. EXAFS R-space experimental curve and fitting curve against the model of Pt-Ru(0001) (see Supplementary Fig. 21) for Pt_1/RuO_2-H. The fit parameters are shown in Supplementary Table 1.

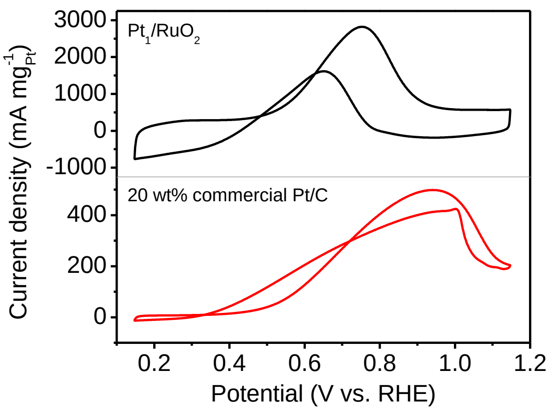

Supplementary Figure 18. Ethanol oxidation of Pt_1/RuO_2 and commercial Pt/C in alkaline media.

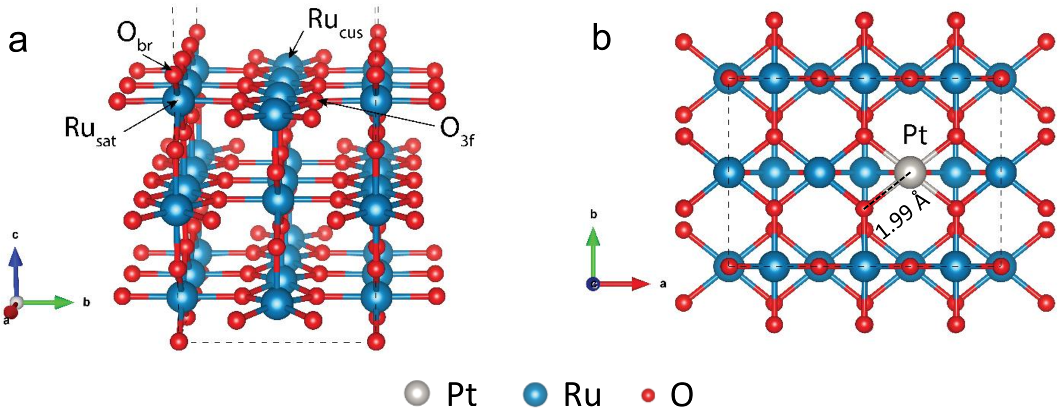

Supplementary Figure 19. Slab models of (a) stoichiometric $RuO_2(110)$, and (b) Pt-$RuO_2(110)$, where a single Pt atom substitutes the Ru_{cus} in $RuO_2(110)$.

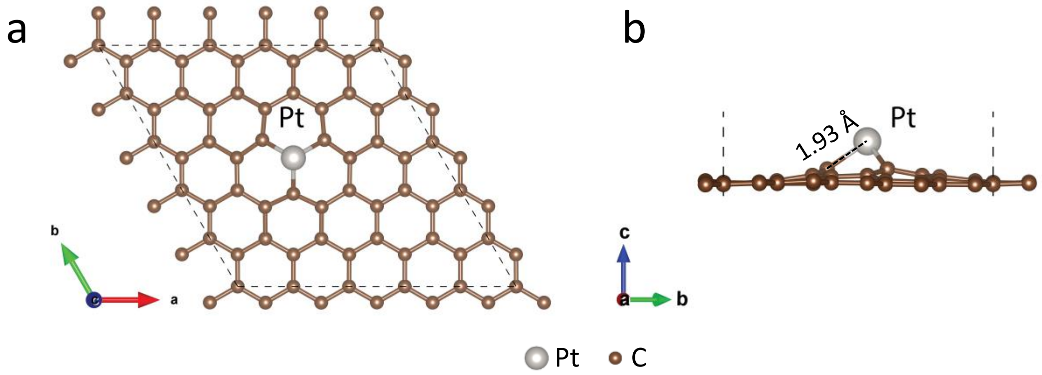

Supplementary Figure 20. PtC3 model corresponding to Pt_1/VXC-72. (a) Top and (b) side view of PtC3.

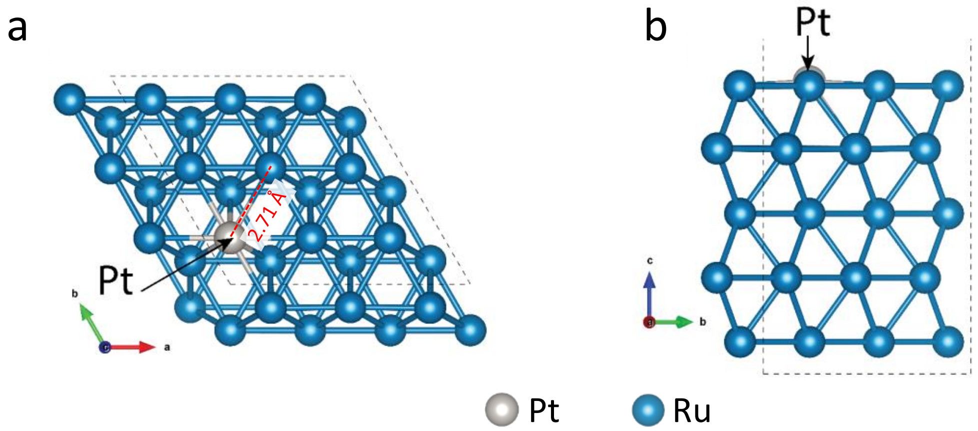

Supplementary Figure 21. Pt-Ru(0001) model corresponding to Pt_1/RuO_2-H. (a) Top and (b) side view of Pt-Ru(0001).

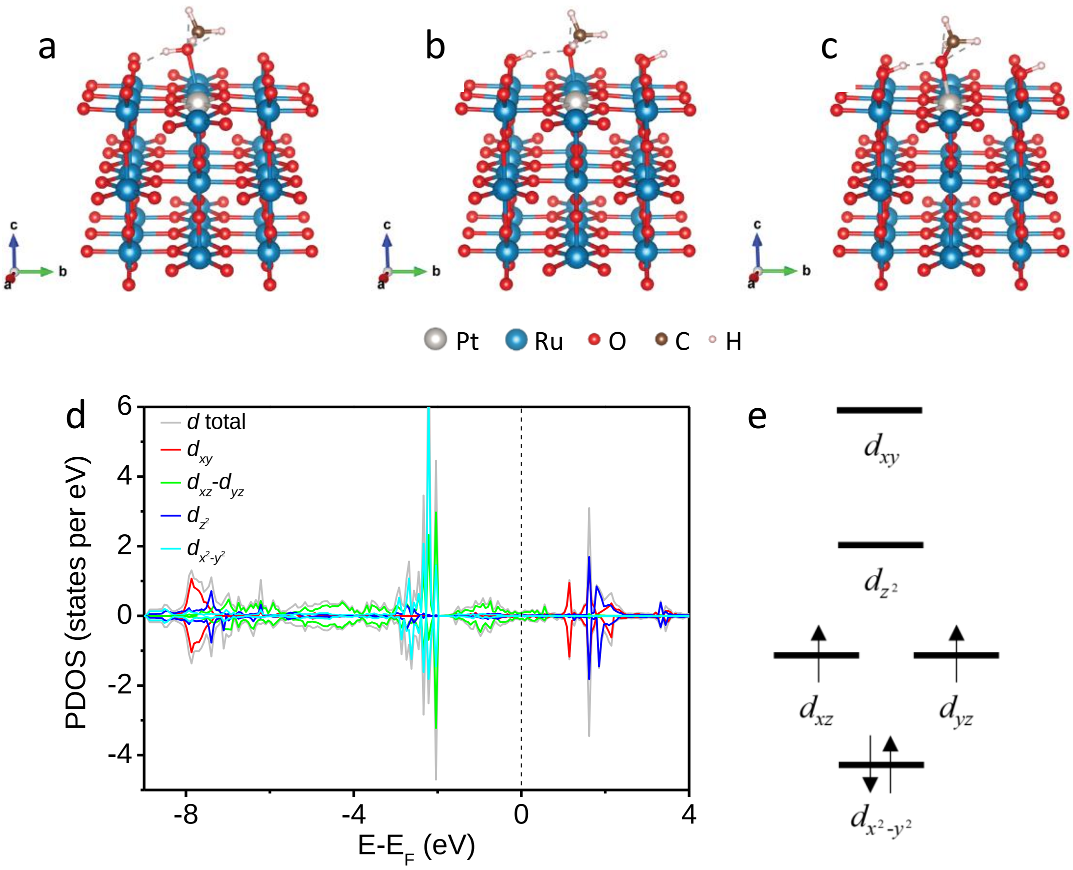

Supplementary Figure 22. The relaxed structures for (a) the adsorption of CH_3OH molecule at the Ru_{cus} site, (b) the co-adsorption of CH_3O and H at the Ru_{cus} site, and (c) the adsorption of CH_3OH molecule at the Pt site on Pt-RuO_2(110). (d) PDOS for Pt atom in the structure as shown in (c). (e) Electronic occupation of Pt corresponding to (d).

The CH_3OH molecule prefers adsorbing above the Ru_{cus} site with a free energy of –0.54 eV. The structure of one CH_3OH molecule at the Pt site by forming the Pt–O bond was also investigated. After the full geometry optimization, the neighboring O_{br} atom attracts the H atom bonded with O in the CH_3OH and breaks the O–H bond ($CH_3OH^* \rightarrow CH_3O^* + H^*$), see Supplementary Fig. 22c. As a result, the CH_3OH molecule can not be adsorbed at the Pt site with its integrity. The breaking of O–H bond during the structure relaxation may be attributed to the strong repulsion between Pt(III) and the adsorbate CH_3OH (Supplementary Fig. 27). After CH_3OH adsorbs at the Pt site of Pt-RuO_2(110) (Supplementary Fig. 22c), the single electron at the dz^2 orbital (Supplementary Fig. 25a) was squeezed out (Supplementary Fig. 22d), leading to the oxidation of Pt(III) to Pt(IV) and deprotonation of CH_3OH^* to form the CH_3O^*. Considering that the energetically unfavorable process of pushing one electron at the dz^2 orbital of Pt to RuO_2 is required to ensure the coordination of CH_3OH at the Pt site, the co-adsorption of CH_3O^* and H^* at Pt site is less stable than that at the Ru_{cus} site by 0.12 eV.

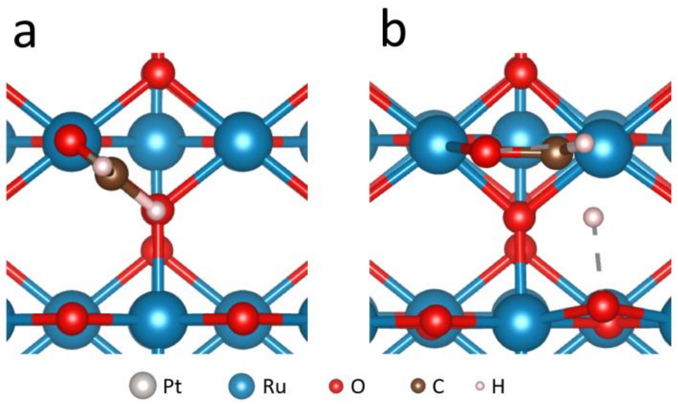

Supplementary Figure 23. The fully optimized (a) initial state and (b) transition state for the reaction of $CH_2O^* \rightarrow CHO^* + H^*$ on the pure $RuO_2(110)$.

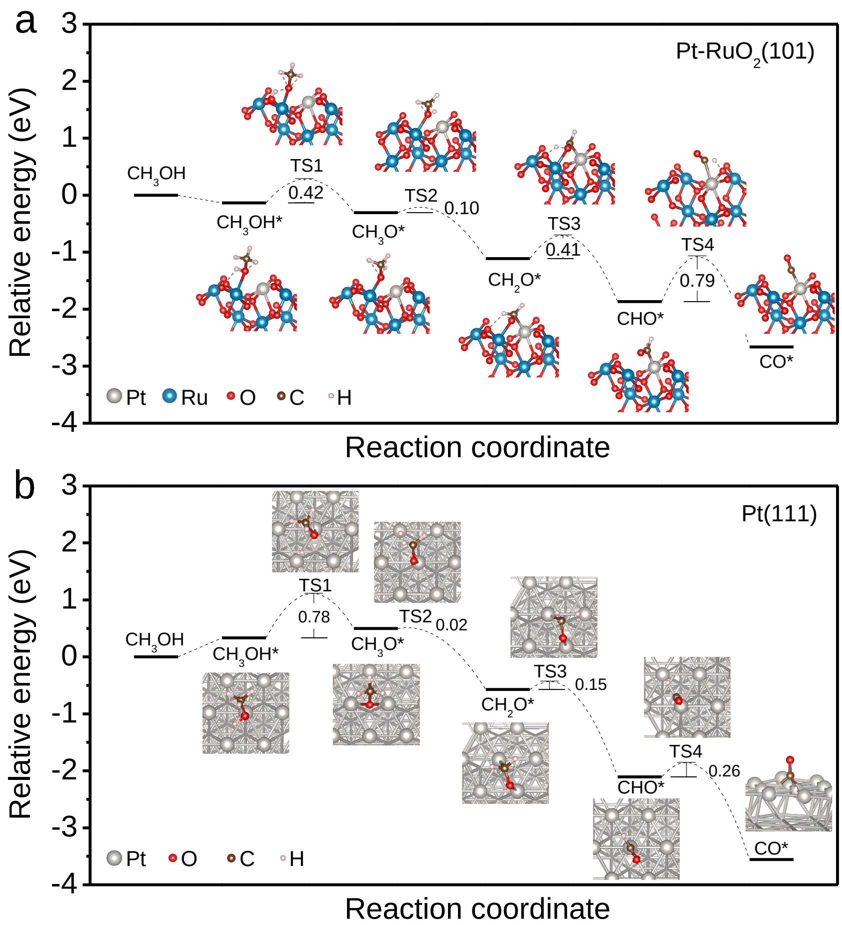

Supplementary Figure 24. Calculated reaction free energy and energy barriers for methanol oxidation to CO. (a) Pt-RuO_2(101). (b) Pt(111).

In addition to the MOR mechanism described in the main text, we also considered the following reaction pathway: $CH_3OH \rightarrow CH_3OH^* \rightarrow CH_3O^* \rightarrow CH_2O^* \rightarrow CHO^* \rightarrow CO^*$ on Pt-RuO_2(101) and Pt(111). The reaction pathway and calculated reaction barriers for Pt(111) are also consistent with the literature[5]. The highest reaction barrier for methanol towards CO on Pt-RuO_2(101) was predicted to be 0.79 eV, a value higher than that on Pt-RuO_2(110) (0.49 eV, Fig. 6a) but much lower than that on PtC3 (2.06 eV, Fig. 6b) and Pt-Ru(0001) (1.08 eV, Fig. 6c). These results suggest Pt_1/RuO_2 favors methanol oxidation and is superior to Pt_1/VXC-72 and Pt_1/RuO_2-H.

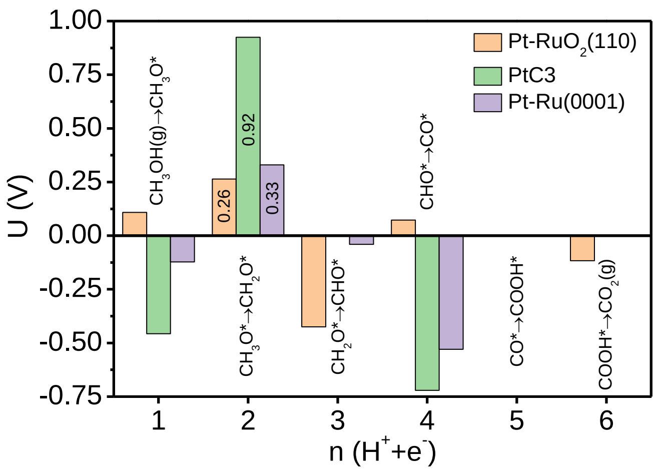

Supplementary Figure 25. The electrochemical potential required for each elementary step towards methanol oxidation at different substrates. The *x*-axis represents total electrons transferred from the original reactant of methanol.

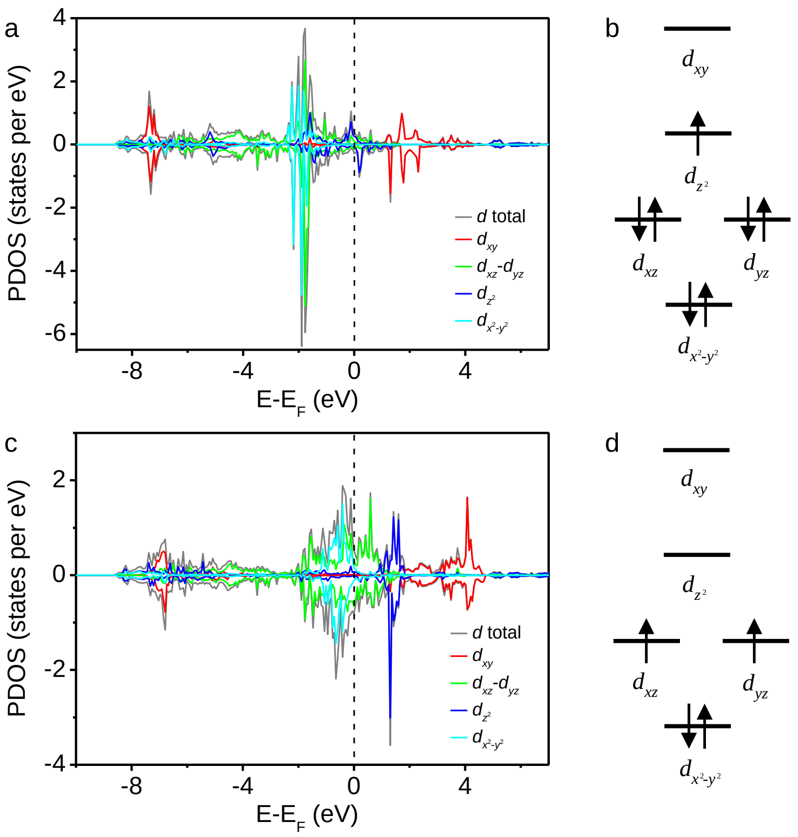

Supplementary Figure 26. PDOS of *d* orbital for (a) Pt and (c) Ru_{cus} in Pt-RuO_2(110) together with the corresponding electronic occupation for (b) Pt and (d) Ru_{cus}.

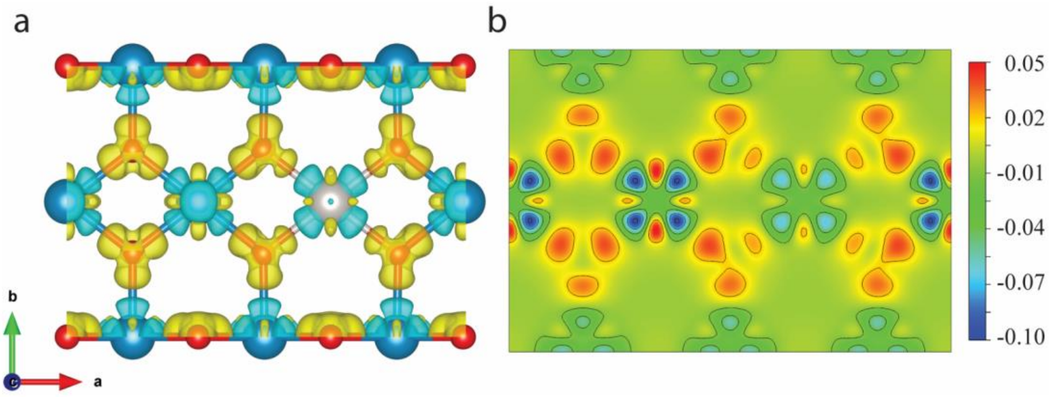

Supplementary Figure 27. Charge density difference for the Pt-RuO_2(110) with respect to the superposition of atomic spheric charge. (a) The topmost atomic layer is shown together with the 0.015 |e|/$Bohr^3$ iso-surface, (blue, grey, and red spheres represent Ru, Pt, and O, respectively). Yellow and cyan colors indicate electron accumulation and depletion with respect to the corresponding spherical atomic charge, respectively. (b) Contour plot (unit of the charge density is |e|/$Borh^3$) of the topmost surface as in (a).

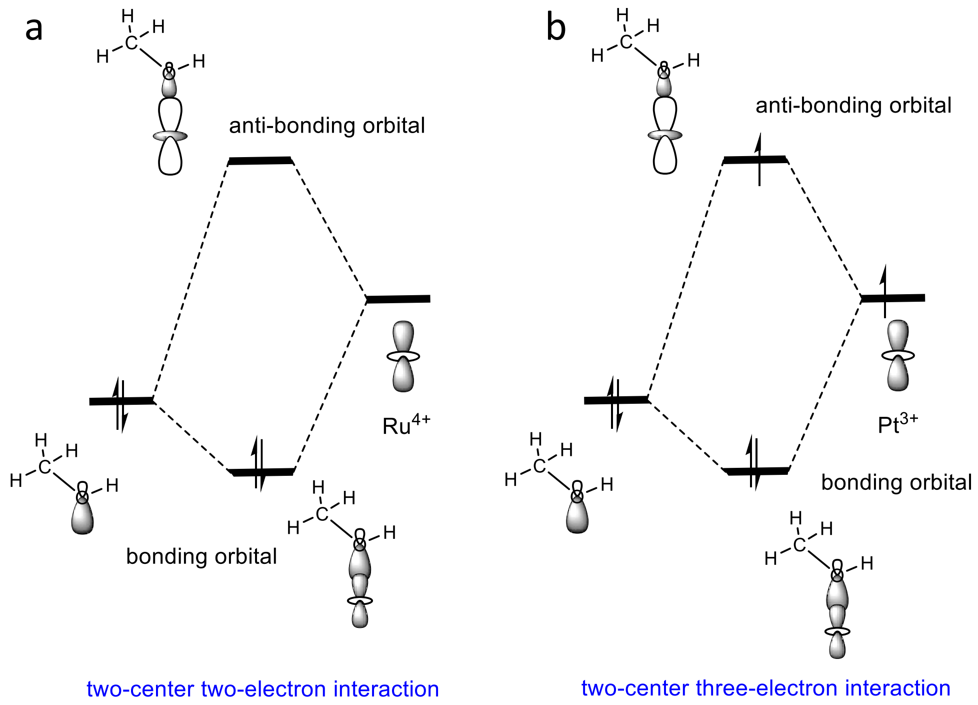

Supplementary Figure 28. Orbital interaction diagrams of methanol with (a) Ru^{4+} and (b) Pt^{3+} in Pt-RuO_2(110). The coordination of the methanol molecule with Ru^{4+} is stronger than with Pt^{3+} due to the more favorable two-center, two-electron interaction of Ru^{4+} compared to the two-center, three-electron interaction of Pt^{3+}.

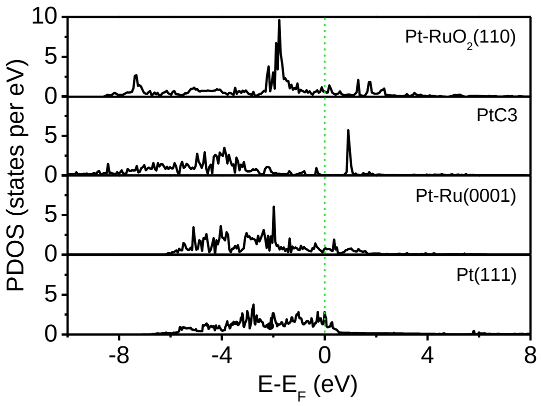

Supplementary Figure 29. PDOS of Pt *d* orbitals in Pt-RuO_2(110), PtC3, Pt-Ru(0001), and Pt(111).

Supplementary Table 1. Structural parameters of Pt_1/RuO_2, Pt_1/VXC-72 and Pt_1/RuO_2-H obtained from EXAFS fitting against Pt-RuO_2(110), PtC3 and Pt-Ru(0001), respectively.

Sample	Shell	N	R (Å)	σ^2 (10^{-3}Å^2)	E_0 (eV)	S_0^2
Pt_1/RuO_2	Pt-O	4.0	1.99	2.30	7.50	0.765
Pt_1/VXC-72	Pt-C	3.0	1.93	1.00	52.0	0.950
Pt_1/RuO_2-H	Pt-Ru	9.0	2.71	8.25	6.00	0.700

Supplementary Table 2. MOR activities of Pt-based electrocatalysts in alkaline media.

Catalyst	Electrolyte	Sweep rate (mV s^{-1})	Mass activity (mA mg_{Pt}^{-1})	Reference
**Pt_1/RuO_2**	0.1M KOH + 1M MeOH	50	6766	This study
$Pt/Ni(OH)_2/rGO$	1M KOH + 1M MeOH	50	1236	6
Pt/CNTs + $CeO_2 \cdot xH_2O$	1M KOH + 1M MeOH	20	2304	7
PtCu NFs	0.5M KOH + 1M MeOH	50	2260	8
Pt-Co nanoframes	1M KOH + 1M MeOH	50	4280	9
$Pt_{56}Cu_{28}Ni_{16}$	1M KOH + 1M MeOH	50	5600	10
Pt_2Bi	1M KOH + 1M MeOH	50	4611	11
PtZn/MWNT	0.1M KOH + 0.5M MeOH	50	~550	12
PtCu-O EONDs	0.5M KOH+ 1M MeOH	50	4430	13
$Pt@HfS_x/CNT$	0.1M KOH + 1M MeOH	50	34.86	14
$Pt-Ce(CO_3)OH/rGO$	1M KOH + 1M MeOH	50	1477.5	15
PtRu nanodendrites	1M KOH + 1M MeOH	50	3060	16
PtNi/C	1M KOH + 1M MeOH	50	~4025	17
$p-Pt_1Cu_1/AP-GNPs$	0.1M KOH + 0.5M MeOH	50	3611	18
Porous Pt nanotubes	1M NaOH + 1M MeOH	50	2331	19
$Pt/Ti_{0.5}Cr_{0.5}N/G$	0.1M KOH + 1M MeOH	50	1068.8	20
AgAu@Pt nanoframes	0.2M KOH+ 1M MeOH	50	483.1	21
Pt_2Bi Nanoplates	1M NaOH + 1M MeOH	50	4820	22

Supplementary Table 3. Reaction free energy for dehydrogenation of CH_3OH and CO electrooxidation on Pt-RuO_2(110) at pH = 13.

Elementary step	ΔG (eV)
CH_3OH + * → CH_3OH*	−0.54
CH_3OH* → CH_3O* + H*	−0.26
CH_3O* + H* + OH^- → CH_3O* + H_2O + e^-	0.17
CH_3O* → CH_2O* + H*	−0.63
CH_2O* + H* + OH^- → CH_2O* + H_2O + e^-	0.14
CH_2O* → CHO* + H*	−1.09
CHO* + H* + OH^- → CHO* + H_2O + e^-	−0.05
CHO* → CO* + H*	−0.51
CO* + H* + OH^- → CO* + H_2O + e^-	−0.15
CO* + OH^- → CO* + OH* + e^-	−0.46
CO* + OH* → *cis*-COOH*	−0.27
cis-COOH* → *trans*-COOH*	0.07
trans-COOH* → *trans*-COOH*	−0.14
trans-COOH* + OH^- → CO_2* + H_2O + e^-	−0.51
CO_2* → CO_2	−0.33

Supplementary Table 4. Calculated Bader charge of selected atoms on Pt-RuO_2(110), PtC3, and RuO_2(110).

Species atom	Pt-RuO_2(110)	PtC3	RuO_2(110)
Pt	+1.22	+0.25	N.A.
Ru_{cus}	+1.61	N.A.	+1.59
Ru_{sat}	+1.83	N.A.	+1.82
O_{br}	–0.72	N.A.	–0.73
O_{3f} connected to Ru_{cus}	–0.89	N.A.	–0.90
O_{3f} connected to Pt	–0.86	N.A.	N.A.
O_{3f} beneath Ru_{cus}	–0.85	N.A.	–0.84
O_{3f} beneath Pt	–0.78	N.A.	N.A.

N.A.: not available.

Note: Pt atom on Pt-RuO_2(110) loses 1.22 e, whereas the Pt on Pt-C loses only 0.25 e. This implies that the Pt on Pt-RuO_2(110) has a higher oxidation state than that on PtC3, which is also consistent with the XANES and XPS results.

Supplementary Table 5. Elementary steps of methanol oxidation that involve electron transfer with corresponding reaction free energy expression.

Elementary step	Reaction free energy
$CH_3OH(g) + * \rightarrow CH_3O* + H^+ + e^-$	$\Delta G_1 = G(CH_3O*) + ½G(H_2(g)) - G(*) - G(CH_3OH(g))$
$CH_3O* \rightarrow CH_2O* + H^+ + e^-$	$\Delta G_2 = G(CH_2O*) + ½G(H_2(g)) - G(CH_3O*)$
$CH_2O* \rightarrow CHO* + H^+ + e^-$	$\Delta G_3 = G(CHO*) + ½G(H_2(g)) - G(CH_2O*)$
$CHO* \rightarrow CO* + H^+ + e^-$	$\Delta G_4 = G(CO*) + ½G(H_2(g)) - G(CHO*)$
$CO* + H_2O(l) \rightarrow COOH* + H^+ + e^-$	$\Delta G_5 = G(COOH*) + ½G(H_2(g)) - G(CO*) - G(H_2O(l))$
$COOH* \rightarrow CO_2(g) + H^+ + e^-$	$\Delta G_6 = G(CO_2(g)) + ½G(H_2(g)) - G(COOH*)$

Note: where $G = E_{DFT} + ZPE - TS$, consistently with the definition for ΔG_{ads} in the main text. Under the standard hydrogen electrode computational framework[23,24], $G(H^+ + e^-)$ is estimated by $½G(H_2(g))$.

References:

1 Yoshida, H., Nonoyama, S. & Hattori, Y. Y. T. Quantitative determination of platinum oxidation state by XANES analysis. *Phys. Scr.*, 813 (2005).

2 Li, Z. *et al.* Iridium single-atom catalyst on nitrogen-doped carbon for formic acid oxidation synthesized using a general host–guest strategy. *Nat. Chem.* **12**, 764-772 (2020).

3 Chen, Y. *et al.* Engineering the atomic interface with single platinum atoms for enhanced photocatalytic hydrogen production. *Angew. Chem. Int. Ed.* **59**, 1295-1301 (2020).

4 Liu, M. *et al.* Atomically dispersed metal catalysts for the oxygen reduction reaction: synthesis, characterization, reaction mechanisms and electrochemical energy applications. *Energy Environ. Sci.* **12**, 2890-2923 (2019).

5 Greeley, J. & Mavrikakis, M. A first-principles study of methanol decomposition on Pt(111). *J. Am. Chem. Soc.* **124**, 7193-7201 (2002).

6 Huang, W. *et al.* Highly active and durable methanol oxidation electrocatalyst based on the synergy of platinum–nickel hydroxide–graphene. *Nat. Commun.* **6**, 10035 (2015).

7 Li, Z.-Y. *et al.* Hydroxyl-rich ceria hydrate nanoparticles enhancing the alcohol electrooxidation performance of Pt catalysts. *J. Mater. Chem. A* **6**, 2318-2326 (2018).

8 Zhang, Z. *et al.* One-pot synthesis of highly anisotropic five-fold-twinned PtCu nanoframes used as a bifunctional electrocatalyst for oxygen reduction and methanol oxidation. *Adv. Mater.* **28**, 8712-8717 (2016).

9 Chen, S. *et al.* High-performance Pt–Co nanoframes for fuel-cell electrocatalysis. *Nano Lett.* **20**, 1974-1979 (2020).

10 Huang, J. *et al.* PtCuNi tetrahedra catalysts with tailored surfaces for efficient alcohol oxidation. *Nano Lett.* **19**, 5431-5436 (2019).

11 Wang, X. *et al.* Bismuth oxyhydroxide-Pt inverse interface for enhanced methanol electrooxidation performance. *Nano Lett.* **20**, 7751-7759 (2020).

12 Qi, Z. *et al.* Sub-4 nm PtZn intermetallic nanoparticles for enhanced mass and specific activities in catalytic electrooxidation reaction. *J. Am. Chem. Soc.* **139**, 4762-4768 (2017).

13 Wu, F. *et al.* PtCu–O highly excavated octahedral nanostructures built with nanodendrites for superior alcohol electrooxidation. *J. Mater. Chem. A* **7**, 8568-8572 (2019).

14 Mayilvel Dinesh, M., Huang, T., Yao, S., Sun, G. & Mao, S. Hafnium sulphide-carbon nanotube composite as Pt support and active site-enriched catalyst for high performance methanol and ethanol oxidations in alkaline electrolytes. *J. Power Sources* **410-411**, 204-212 (2019).

15 Chen, G. *et al.* Synergistic effects of platinum–cerium carbonate hydroxides–reduced graphene oxide on enhanced durability for methanol electro-oxidation. *J. Mater. Chem. A* **7**, 6562-6571 (2019).

16 Guo, K., Liu, Y., Han, M., Xu, D. & Bao, J. Highly branched ultrathin Pt–Ru nanodendrites. *Chem. Commun.* **55**, 11131-11134 (2019).

17 Lu, S., Li, H., Sun, J. & Zhuang, Z. Promoting the methanol oxidation catalytic activity by introducing surface nickel on platinum nanoparticles. *Nano Res.* **11**, 2058-2068 (2018).

18 Zhang, G., Yang, Z., Zhang, W. & Wang, Y. Facile synthesis of graphene nanoplate-supported porous Pt–Cu alloys with high electrocatalytic properties for methanol oxidation. *J. Mater. Chem. A* **4**, 3316-3323 (2016).

19 Lou, Y. *et al.* Porous Pt nanotubes with high methanol oxidation electrocatalytic activity

based on original bamboo-shaped Te nanotubes. *ACS Appl. Mater. Interfaces* **8**, 16147-16153 (2016).

20 Liu, B., Huo, L., Si, R., Liu, J. & Zhang, J. A general method for constructing two-dimensional layered mesoporous mono- and binary-transition-metal nitride/graphene as an ultra-efficient support to enhance its catalytic activity and durability for electrocatalytic application. *ACS Appl. Mater. Interfaces* **8**, 18770-18787 (2016).

21 Yan, X. *et al.* Triangular AgAu@Pt core–shell nanoframes with a dendritic Pt shell and enhanced electrocatalytic performance toward the methanol oxidation reaction. *Nanoscale* **10**, 2231-2235 (2018).

22 Yuan, X. *et al.* Intermetallic PtBi core/ultrathin Pt shell nanoplates for efficient and stable methanol and ethanol electro-oxidization. *Nano Res.* **12**, 429-436 (2019).

23 Nørskov, J. K. *et al.* Origin of the overpotential for oxygen reduction at a fuel-cell cathode. *J. Phys. Chem. B* **108**, 17886-17892 (2004).

24 Ferrin, P. & Mavrikakis, M. Structure sensitivity of methanol electrooxidation on transition metals. *J. Am. Chem. Soc.* **131**, 14381-14389 (2009).
